# Barriers to Care Encounter: A Model That Empowers Underserved Populations and Promotes Cross-Cultural Preparedness in Medical Students

**DOI:** 10.15766/mep_2374-8265.11608

**Published:** 2026-06-11

**Authors:** Angelica Nibo, Haley Lewsey, Neeti Swami, Radha Patel, Lauren Cobbs, Fiona Prabhu

**Affiliations:** 1 Alumna, School of Medicine, Texas Tech University Health Sciences Center; 2 Resident Physician, Department of Pediatrics, University of Texas Southwestern; 3 Associate Professor and Senior Associate Dean for Student Affairs, Department of Medical Education, Texas Tech University Health Sciences Center; 4 Professor, Department of Family Medicine, Texas Tech University Health Sciences Center

**Keywords:** Communication Skills, Diversity, Equity, Inclusion, Social Determinants of Health, Simulation, Standardized Patient

## Abstract

**Introduction:**

While identifying and interacting with patients adversely impacted by social determinants of health (SDoH) and related barriers to care is essential in medical education, current educational models lack meaningful patient involvement and feedback. This project aims to implement an activity focused on barriers to care that incorporates and empowers representative standardized patients (SPs) to share their lived experiences with students.

**Methods:**

Forty-five first- and second-year preclinical medical students participated in a 90-minute intervention including a lecture; a prebrief; a simulated clinical encounter with a patient who was nonadherent to treatment because of a financial, social, or cultural barrier; and a debrief. All SPs had personal experiences with barriers to care. This intervention was implemented in January and October of 2024 and evaluated via analysis of pre- and postencounter Likert surveys that were administered to students.

**Results:**

In January, statistical analysis of pre- and postsurveys indicated a significant increase in participants’ reported ability to elicit a comprehensive history and enhance patient adherence (*P* < .05). After content revision, analysis of the October cohort demonstrated significant increases in reported ability to describe historical and systemic impacts, elicit a comprehensive history, enhance adherence, and understand own biases (*P* < .05).

**Discussion:**

The Barriers to Care Encounter was effective in increasing student knowledge, skills, and attitudes on addressing SDoH in clinical practice while allowing medical students to engage with representative patient populations. Future directions include performing qualitative analyses of SP feedback and comparing impact of representative SP encounters to nonrepresentative SP encounters.

## Educational Objectives

By the end of this activity, learners will be able to:

1. Describe the cultural background, economic factors, and social factors of a patient and how these affect the patient's health care decisions.
2. Recognize how to elicit a cultural, social, and medical history to assess a patient's adherence using open-ended and affirming communication strategies, reflective listening, and summarization based on the patient's values to increase rapport.
3. Identify negotiation skills needed to enhance a patient's adherence despite their barriers to receiving quality health care.
4. Value the application of social determinants of health to patient care.
5. Discuss the impact of their cultural backgrounds and biases on their interactions with people from different backgrounds.

## Introduction

Social determinants of health (SDoH) are defined in public health as social systems and the resources and hazards these systems influence that impact health and health trends by demographic.^1^ Hazards and inaccessible resources create barriers to care and health disparities, or systemic differences in health outcomes and SDoH in specific populations.^2^ One such hazard is social bias: prejudice or discrimination directed at a person, group, or set of beliefs, including racial or ethnic minorities, women, LGBTQ+ people, among others.^3^ To illustrate, social bias, such as a belief that Black and Hispanic patients have a higher pain tolerance or exaggerate symptoms, is a hazard associated with fewer pain medication prescriptions and lower dosages than White patients with similar injuries.^3^

In US undergraduate medical education, curricula are required by the Liaison Committee on Medical Education (LCME) to include instruction on cultural competence.^4^ There is extensive literature detailing the structure, content, and evaluation of these curricula, often including SDoH, barriers to care, and health disparities. Programs that apply barriers to care to real-life scenarios range in implementation style from case-scenario discussions to role-play in a classroom setting to formal standardized patient (SP) encounters.^5–13^ One *MedEdPORTAL* publication by Burke et al.^5^ presents a workshop teaching medical students how to identify a patient's barriers and offer resources, with case studies for practice. Another *MedEdPORTAL* curriculum by Howell et al.^6^ describes role-play in which students collect social history from a classmate acting as a patient experiencing barriers.

While these programs fit LCME criteria and demonstrate efficacy in improving students’ understanding of barriers to care, they rarely include engagement with real patients. We acknowledge the inherent challenge in studying the relationship between a medical school educational intervention and a patient's health care experience years later. Patient experiences are shaped by multiple factors beyond a single educational interaction, just as physicians’ approaches to SDoH are influenced by explicit and implicit lessons throughout training and practice. Some curricula include local community members with lived experience of barriers to care. For example, Symons et al.^8^ describe a program in which students interact with individuals with disabilities and their families in classroom and community settings, and complete standardized encounters in which people with disabilities are trained as SPs. Additionally, Berger and Harada^14^ recruit community members with histories of myocardial infarction for student interviews and neighborhood exploration to better understand how SDoH affect their care. While these approaches incorporated representative voices into medical education, a gap remains such that few publications include feedback from those community members to inform students’ learning and development.^15,16^

In response, we designed a nongraded SP encounter for preclinical students involving representative actors experiencing simulated barriers to care. We aimed to address gaps in students’ knowledge of historical and systemic impacts of health disparities, while developing their skills in eliciting holistic, culturally sensitive histories, facilitating treatment adherence, and emphasizing the value of SDoH and understanding one's own bias. In developing this approach, we drew on prior work. Woodard et al.^15^ describe an approach in which volunteers with disabilities served as “model patients” guiding students in history-taking and physical exam, and participated in postencounter discussions. Similarly, Green et al.^16^ implemented a standardized encounter based on real events and recruited bilingual, Latina SPs, who provided feedback to students from the perspective of a Spanish-speaking Latina. Our approach builds on and extends this work through a unique instructional approach. We recruit SPs with lived experience of barriers to care and develop multiple individualized cases to guarantee each SP's case is true to their personal experience. This method has an innovative interface between SPs and students. We allow students to receive feedback from an SP who has directly faced the barrier, distinct from Green et al.,^16^ without requiring the SPs to speak about sensitive personal details, distinct from Woodard et al.^15^ In contrast to these prior models, our approach balances authenticity with psychological safety, offering a meaningful learning experience while mitigating ethical concerns associated with asking individuals to recount personal health care experiences.

## Methods

### Curriculum Development

Using Kern's 6-step approach to curriculum development, our first step (problem identification) was to identify that preclinical medical students lacked knowledge on addressing barriers to care despite health impact.^20^ Following step 2 of Kern's approach (Targeted Needs Assessment), curriculum review of our institution revealed a lack of preclinical education on identifying and addressing barriers to care. We then followed the remaining 4 steps to build, implement, and evaluate our curriculum.

We selected hypertension and asthma for our case presentations due to their high prevalence and student familiarity. We also included diabetes as a comorbid condition to increase potential for financial barriers. The hypertension case's clinical features and management followed the American Academy of Family Physicians’ (AAFP) description of hypertension with early end-organ damage.^17^ The asthma case, derived from AAFP description of mild intermittent asthma with added comorbid insulin-dependent diabetes, allowed volunteers under age 25 years to portray a patient requiring multiple medications in their age range rather than portraying an older patient.^18^

### Curriculum Implementation

Based on Kolb's learning theory, which emphasizes experiential learning and self-reflection, we conducted this intervention in 2 parts: lecture and SP interaction (Appendix A) followed by a reflective debrief.^19^ This also reflected elements of constructivist learning theory, including building upon prior learning, as students received initial background via lecture and then conducted an SP encounter followed by a reflective discussion session.^19^ For each student, the duration of the entire intervention was 90 minutes. Prior to intervention, all participants were preclinical medical students who had received training in gathering a basic patient history. To encourage student participation, we conducted a session in January 2024 as elective credit for the Clinical Practice with Underserved Populations (CPUP) Elective at Texas Tech University Health Sciences Center School of Medicine (TTUHSC SOM), which included first- and second-year medical students. We later offered an October 2024 encounter as a for-credit session during a class-wide enrichment week, alongside multiple other events, for first-year medical students.

All but 1 student who participated in the SP interaction attended a large group lecture on motivational interviewing in the context of barriers to care and SDoH (Appendix B). SP interactions took place at the institutional simulation center and required private rooms for conversation. Up to 6 simulation rooms operated simultaneously, contingent on time slot occupancy. We informed students to wear scrubs or business casual with white coats. In January, students completed their encounter one-on-one with their patient. In October, due to limited resources, groups of 2 to 3 students performed their encounters.

Prior to the SP encounter, students received instructions (Appendix C) with a patient profile and review of the OARS model for motivational interviewing, which stands for Open-ended questions, Affirmations, Reflections, and Summaries.^21^ We provided students a template (Appendix D) for gathering a patient history and informed them that the patient was facing 1 or more barriers to health care.

During a 5-minute prebrief, students completed a presurvey (Appendix E) and reviewed preencounter instructions with a coordinator. Then, students gathered a patient history over 15 minutes while using the OARS model to identify barriers. Afterward, SPs gave verbal feedback to students concerning their comfort during the interaction. We did not use the skills checklist (Appendix F) in our evaluation of students, and it primarily served to provide SPs with reference points during their verbal feedback. The debrief followed, during which students spent 30–45 minutes discussing their experience with a coordinator, guided by discussion questions (Appendix G) and projected slides (Appendix H). We employed a learner-centered approach to build on students’ prior knowledge and accommodate diverse learning styles.^19^ Based on student feedback from January's session, we expanded the October presentations (see Appendices B and H) and invited social workers as guest speakers. After all activities, students completed the postsurvey (Appendix I). The pre- and postsurveys consisted of Likert scales comparing participants’ grasp of the educational objectives before and after the encounter through knowledge, skills, and attitudes domains and allowed students to provide feedback.

### Volunteer Roles and Preparation

Volunteer roles consisted of the preencounter, encounter, and postencounter coordinators and the SPs. The coordinators were medical student volunteers who met prior to the event to assign roles, review related materials, and understand the flow of activities. The preencounter coordinator oriented students upon arrival, ensured completion of the presurvey, and reviewed prebrief instructions. The encounter coordinator gave warning knocks to alert students when 5 minutes remained in their encounter and when to switch to SP feedback, and they helped transition students into and out of their simulation rooms. The postencounter coordinator led the debrief and ensured students completed the postsurvey.

We recruited SPs through TTUHSC SOM's existing paid SP pool and flyers shared with community members and undergraduate student organizations (Appendix J). All SPs met the following criteria: age 18 years or older and personal experience with at least 1 barrier to care. We individually emailed each SP for a story describing their experience. A coordinator embedded 1 applicable story and barrier into the context of chronic hypertension or asthma. We emailed this social history to the SP to verify their comfort and that the depiction was accurate to their experience. Once we received SP approval, we sent the complete case (Appendix K) and the communication skills checklist (see Appendix F) to the SP for preparation for their role, resulting in 7 cases with individualized social histories. Coordinators reviewed the cases, and SPs reviewed and revised the barriers section. Based on our SPs’ experiences, we included the following barriers: distrust of the health care system, cost of care, insurance coverage, transportation, inflexible work schedules, and cultural/language barriers. This list was not exhaustive; curriculum developers may incorporate additional barriers based on selected SPs’ experiences.

## Results

Forty-five students participated in this encounter: 14 in the January session and 31 in the October session. All students completed the presurvey, whereas 12 (86%) of the January group and 22 (71%) of the October group completed the postsurvey.

Our analysis supports an upward trend in students’ perceived competence in most of our educational objectives. After the encounters, we performed 1-tailed *t* tests of equal variance to compare the pre- and postsurvey for each group. This measured whether there was a significant increase in perceived competence from pre- to postsurvey for each objective within each student group. We found a significant increase in participants’ agreement with ability to “elicit a cultural, social, and medical history” and “assess and enhance patient adherence” in both the January group and the October group (Table). Furthermore, the October group saw a significant increase in participants’ agreement that they could describe “the historical impact of racism and cultural issues in relation to current health disparities” and “systemic and medical encounter issues” (see Table). Neither group showed a significant change in valuing SDoH, as 100% of participants agreed with the statement before and after the encounter (see Table).

**Table.**
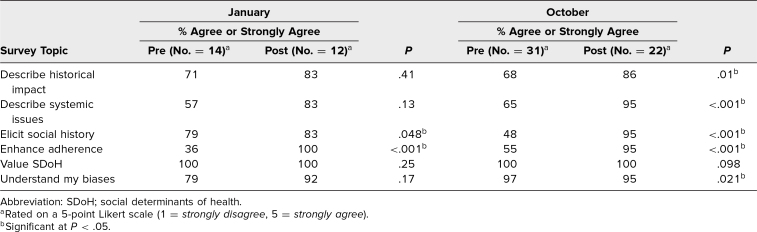
Change in Medical Student Agreement Across Survey Topics

## Discussion

The Barriers to Care Encounter was developed as a simulation that teaches preclinical students to explore the cross-cultural, individual, and structural circumstances that can impact patient care. In January, we found general upward trends in reported abilities, but the significance was uncertain, possibly due to the small sample and/or the need for improvement, such as further discussion of how to connect clinical realities to historical events and counteract biases with open-minded thinking. Subsequently, with revisions and a larger sample, the October intervention demonstrated greater confidence in its effectiveness. For medical education, our intervention represents progress toward demonstrating the combined impact of teaching the roots of SDoH, teaching skills for socially conscious communication, and hearing patient input on students’ preparedness for encounters involving barriers to care.

Our unique patient input method enabled SPs to guide simulation development and then bring forth gaps in our students’ performance. Our data suggest that students’ perceived ability to explore treatment nonadherence increased after gaining a better understanding of the sociohistorical factors affecting care and of what their patients were seeking in their care. Yet, there are very few studied SDoH curricula in which patient-centered assessment affects students’ evaluations.^22^ A student cannot accurately evaluate their skills for communicating with target populations or navigating barriers without asking the target patients how this communication was received. Therefore, the patient perspective informs efficacy. This intervention bridged the gap in the ability for undergraduate medical trainees to receive feedback from patients by having students listen to their SPs share their experience with “othering,” or creating distance between themselves and the patient, and how to avoid it.^23^

We discovered that the greatest limiting factor for the development phase was the SP recruitment process. Only a few SPs from the university's pool had experience with barriers. Consequently, we printed recruitment flyers for underserved community organizations and the nearby undergraduate campus cultural clubs. By connecting with local organizations, schools can foster new relationships with the community, especially if the encounter is repeated annually. Community partnership aligns with this study's scope, creating preclinical interactions with patients with lived barriers, by cultivating an activity that realistically resembles what students will experience if they go on to serve the needs of this local community's patients and by promoting health care education that reflects the needs of the university's surrounding community. To improve recruitment, we recommend that future developers attend multiple events hosted by target organizations over 2–3 months to build a bond, check in serially with selected patients, and find more volunteers.

For the January pilot, our simulation structure and coordinator roles enabled efficient transitions and timekeeping, and our partnership with undergraduate pre-medical student volunteers enabled networking with medical students and further interest in the medical school. However, we found the debrief insufficient for the depth of knowledge and attitude concepts that coordinators and students wanted to discuss. Thus, the next academic year, we extended the debrief session content and invited social workers to share their expertise and resources. Additionally, we expanded implementation to a class-wide course to allow the October encounter to become an option to all first-year medical students, which increased participation and revealed that course type may have contributed to a low sample size in January. Once encounters ended, displaying the postsurvey QR code in the debrief session allowed us to gather immediate feedback from students. Students generally found the experience valuable, but 1 student from the January cohort suggested a greater focus on attitudes during pre- and postdiscussions. In response, we restructured these discussions to devote time to teaching open-minded responses, displaying respect when cultural experiences conflict, and demonstrating humanity and allyship in clinical encounters.

Although the intervention appears to address educational needs for addressing health disparities, reliance on self-reports may fall short of accurately reflecting the extent to which these needs were met. This was augmented by input from our SPs, who provided representative external perspectives to confirm or challenge students’ self-beliefs. Immediately after the January and October encounters, a coordinator met face-to-face with the SPs to ask open-ended questions about their experiences that were not prewritten or standardized for data analysis, but were used to gather informal feedback beyond the students’ self-reports. In January, the SPs perceived the students as “really good future doctors” but noted missed opportunities to ask follow-up questions, nonverbal cues unintentionally communicating discomfort, limited familiarity with local resources, and potential for students to further convey that “they're on [the patient's] side.” The SP feedback was used to create a new prebrief presentation for the October encounters, which included scenario-based discussion slides focused on motivational interviewing, timing for eliciting barriers, local resources, and empathetic responses and actions (see Appendix B). In October, the SPs noted that while several students searched for resources on their phones, some again focused too heavily on symptoms, and each patient name could be changed to match the cultural background of each recruited SP (e.g., Farsi). This suggests that the prebrief could better reinforce the intentional facilitation of conversations about home life, affording rent and medications, and cultural practices.

Limitations of this intervention include the impact of state legislation on funding and the extent of simulation resources available. This encounter was subject to Texas legislation, which reduced funding available to the CPUP Elective. This limited our ability to compensate participating patients but was mitigated by informed recruitment of nonpaid volunteers. The institution's simulation space and availability will determine scalability. Evaluation limitations include differences between the January and October implementations, sample size, reliance on self-reports, and the lack of standardized SP feedback. While the modification of the October intervention introduced variables (students having partners and scenario-based discussion), which prevented a combined (January-October) analysis, it allowed for meaningful data on the effect of these modifications. However, our data lacks external validation and assessment of the long-term impact, which could be improved through qualitative analysis of SP feedback and follow-up with students.

In conclusion, the Barriers to Care Encounter is beneficial to cross-cultural and SDoH education as a generalizable patient simulation that focuses on specific barriers and promotes representation. Our use of Kolb's and constructivist learning theories appeals to differing learning styles by encouraging progressive learning through reflection and experience.^19^ Other institutions can adapt this simulation for other barriers, unique local health disparities, implementation with third- and fourth-year students, or integration with basic science curriculum simulations. In the future, developers could benefit from extending SP recruitment and conducting a thematic analysis of SPs’ evaluations of students. This would provide insight into the abstract inadequacies with these interventions that patients feel meaningfully impact their care but are not quantitatively measurable. Given the paucity of patient input in the literature, a thematic analysis could determine if themes identified as concerns for patients reflect or add to the themes often cited as important to students and faculty, such as avoidance of stereotyping/bias and reflexive thinking.^22,23^ Additionally, this enables ethical solicitation of patient feedback by providing a safe simulated setting, removed from the clinical setting, for patients to discuss sensitive themes with students. Finally, curriculum developers can compare responses to cultural simulations with non-representative actors against those with representative actors for all programs seeking to study the effect of representation on cross-cultural education.

## Appendices


SP Case.docxLecture and Prebrief.pptxStudent Preencounter Instructions.docxStudent Guide for Gathering a History.docxPreencounter Survey.docxCommunication Skills Checklist.docxDebrief Discussion Questions.docxPostencounter Debrief Presentation.pptxPostencounter Survey.docxRecruitment Flyer.docxCase Overview and SP Training.docx
*All appendices are peer reviewed as integral parts of the Original Publication.*
