# Supplementary material for: Barriers to Care Encounter: A Model That Empowers Underserved Populations and Promotes Cross-Cultural Preparedness in Medical Students
Source: MedEdPORTAL. 2026 Jun 11;22:11608. doi: 10.15766/mep_2374-8265.11608 (PMC13253653; doi:10.15766/mep_2374-8265.11608)
Supplement: Supplementary file 1 — SP Case.docxLecture and Prebrief.pptxStudent Preencounter Instructions.docxStudent Guide for Gathering a History.docxPreencounter Survey.docxCommunication Skills Checklist.docxDebrief Discussion Questions.docxPostencounter Debrief Presentation.pptxPostencounter Survey.docxRecruitment Flyer.docxCase Overview and SP Training.docx [file mep_2374-8265.11608-s001.zip › A. SP Case.docx]

**Appendix A: Standardized Patient Case**

Date: 10/11/2024

Primary Case Author: Angelica Nibo

Secondary Case Author: Radha Patel

Standardized Patient Educator: Angelica Nibo

Name of Case: Nonadherence Due to Barriers to Care

Name of Educational and/or Assessment Activity: Motivational Interviewing/Pre-Encounter Lecture and Pre- and Post-Encounter Survey

Patient Name: Maya/Michael Willow

Chief Complaint: High blood pressure / difficulty breathing

Most Likely Diagnosis and Differential With Rationale From History and/or Physical Exam: Hypertension / Asthma

Challenge Question: What barriers are preventing your patient from receiving quality care?

Domains: Check all that apply

- Professionalism
- Communication and Interpersonal Skills
- Medical History
- Physical Exam
- Shared Decision-Making
- Patient Education
- Clinical Reasoning
- Documentation
- Handoff
- Presentation
- Other:

Type and Level of Learner: First- and second-year medical students

Case Objectives (for both cases): Please list specific objectives for each of the domains you have checked above:

1. Describe the cultural background, economic factors, and social factors of a patient and how these affect the patient's healthcare decisions.
2. Recognize how to elicit a cultural, social, and medical history to assess a patient’s adherence using open-ended and affirming communication strategies, reflective listening, and summarization based on the patient's values to increase rapport.
3. Identify negotiation skills needed to formulate a plan to enhance a patient’s adherence despite their barriers to receiving quality healthcare.
4. Value the application of social determinants of health to patient care.
5. Discuss the impact of their cultural backgrounds and biases on their interactions with people from different backgrounds.

| SETTING: outpatient, in patient, ED, home, nursing home, rehab, group, etc. | Outpatient |
| --- | --- |
| PATIENT PROFILE: Information about the “patient” that helps select an SP and helps the learner get an understanding of them as a person. SP will know more information about the patient than learner will ever ask but allows SP to portray a fully developed patient personality. If none of the items below are particulars for the case, please write “all may be used.” | |
| Case | Hypertension follow-up |
| Age range | Age is same as SP’s real age |
| Religious/spiritual background | All may be used (should match SP’s barrier if applicable*) |
| Sex (e.g., male, female, intersex, transwoman, transman) | All may be used (should match SP’s barrier if applicable*) |
| Sexual orientation (e.g., heterosexual, lesbian, gay, bisexual, pansexual, queer, asexual) | All may be used (should match SP’s barrier if applicable*) |
| Gender expression (e.g., man, woman, genderqueer) | All may be used (should match SP’s barrier if applicable*) |
| Race and ethnicity (e.g., to promote educational diversity, we use a diverse pool of SPs.) | To promote educational diversity, we use a diverse pool of SPs |
| Physical description (e.g., BMI, height range) | 5’10” and 190 lbs. (May not match SP) |
| Physical limitations | None |
| Patient appearance (e.g., disheveled, hospital gown, business casual, casual) | Casual |
| Moulage + location (e.g., none, bruises, scars, body piercing, tattoos) | None |
| Affect (e.g., pleasant, cooperative) | Patient should be tired and slightly uncomfortable/standoffish at first due to prior dissatisfaction with the healthcare system/previous providers |
| Family group (e.g., who is family, who they live with) | Spouse. No siblings, children, or pets. |
| Education | Completed high school. |
| Level of health literacy | Average |
| Employment, if any - present and past, noting any current stresses | Works at Market Street Grocery |
| Home/homeless - type of dwelling, number of stories, owned or rented | Living in owned apartment |
| Financial situation - any current stresses | All may be used (should match SP’s barrier if applicable*) |
| Insurance status (e.g., un/under/insured, public/private, HMO/PPO) | All may be used (should match SP’s barrier if applicable*) |
| Habits (i.e., diet, exercise, caffeine, smoking, alcohol, drugs) | None |
| Activities (i.e., hobbies, sports, clubs, friends) | SP answers with their real life hobbies |
| Typical day - what is the usual daily routine | Work, hobbies/spending time with spouse |

| CASE INFORMATION | Hypertension Follow Up |
| --- | --- |
| Chief Concern: What the patient will say when greeted by the student. The patient’s primary reason for seeking medical care often stated in their own words. | “Here to check my blood pressure and breathing” |
| Additional Concerns: Other, if any, concerns the patient has today (i.e., symptoms, requests, expectations, etc.) that will become part of set agenda. | None |
| THE PATIENT’S STORY: The SP will be asked to tell their symptom story and the personal and emotion impact for each of their concerns. You will want to write this in the patient’s voice. The symptom story should be able to answer this question: “Tell me more about [chief concern/additional concern], starting at the beginning and bringing me up to now.”  The personal context should be able to answer questions concerning the broader personal/psychosocial context of symptoms, especially the patient’s beliefs/attributions.  The emotional context should be able to ask how are you doing with this, how does this make you feel, how has this affected you emotionally? IMPACT: How has this affected your life? How has this been for your family? | Story: Came in for a check up with a different doctor last year and was told my blood pressure was really high and now I’m having bad headaches, and a hard time breathing.  (If asked, patient does not check their blood pressure at home.)  Context: (Patient should be tired and slightly uncomfortable/standoffish at first due to prior dissatisfaction with the healthcare system/previous providers. Patient should give little to no detail about personal barriers until after the social history if the student has made them feel comfortable and heard.)  Impact: It makes it hard to focus or concentrate on anything and now I’m getting worried that something’s really wrong with my chest. |
| HISTORY OF PRESENT ILLNESS: Although some of the HPI will be given in the patient’s symptom story, the learners will expand the story during the direct question section. Below, describe the detailed history, usually about the chief concern, which the student must develop in order to make a useful assessment of the problem: | |
| Onset (when; gradual or sudden) | I had a blood pressure of 160/90 about a year ago but the headaches, and difficulty breathing started this week. |
| Setting (what was going on or where was patient when symptoms first noticed?) | Nothing has changed recently and no recent illness.  Not checking blood pressure at home. |
| Duration (how long) | All pain occurs every day and never goes away |
| Time relationships (frequency, constant or intermittent) | Constant |
| Location | Headaches: both temples |
| Radiation | No radiation, the pain just stays around my temples. |
| Quality | The headaches feel like a sharp stabbing pain  Hard to take deep breaths/get enough air in |
| Amount | Headache pain is 7/10.  It’s been hard to breathe all week but sometimes it feels like a 2/10 and other times it feels like a 5/10. It just depends on the day. Today it feels like a 4/10. |
| Aggravated by what | It gets worse when I’m walking or while at work. |
| Relieved by what | Nothing makes it feel better. Tried Tylenol and Ibuprofen with no relief. |
| Associated with what | Some dull chest pain. No coughing, wheezing, fevers, or chills. Has not had an A1C/blood sugar level checked recently. |
| Attitude (what does the patient think is the problem, and how do they feel about it) | I’m getting worried that something’s really wrong with my chest. |
| Overall course | Year long history of hypertension, now with new onset headaches, chest pain, and dyspnea |
| REVIEW OF SYSTEMS: Significant positives and negatives | |
|  | Positive for shortness of breath with sharp pain, chest pain, shortness of breath while lying flat, shortness of breath with exertion, swelling in legs up to the middle of the shin |
|  | Positive for fatigue, weakness, headaches, polydipsia (thirsty all the time) |
|  | Negative for syncope/fainting, vision changes, palpitations |
| Past medical history | Childhood: none  Adult: Type II Diabetes, hypertension  If asked, no history of thyroid, cortisol, or aldosterone issues  Psychiatric: None |
| Medication allergies (name and reaction) | None |
| Environmental allergies (name and reaction) | None |
| Illnesses | None |
| Vaccinations | Up to date |
| Surgeries | None |
| Accidents/injuries/trauma | None |
| Hospitalization | None |
| Inclusive sexual and reproductive history | |
| Sexual practices  Sexual partners  Protection: Use of safer sex practices  Use of birth control if appropriate  Risk of intimate partner violence | 5 lifetime partners, sexually active with spouse, uses contraceptives, no history of STIs, has experienced vaginal sex (answer can be “no intimate sexual experiences” if contradictory with religion). Feels safe and happy with partner. No risk of intimate partner violence. |
| OB/GYN history  Age of onset of menses  Age of menopause  Number of pregnancies  Number of live births  Number of miscarriages  Number of abortions | (if age >45): No previous pregnancies or live births. Last menstrual period was over a year ago. Age of Menopause was 45. No related symptoms.  (If age 18 - 45): No previous pregnancies or live births. Last menstrual period was 2 weeks ago. Periods are regular and monthly, with some cramps. |
| Medications  Prescription/dose/reason  Over the counter/dose/reason  Herbs/supplements/dose/reason  Other: | I was prescribed Lisinopril, Metformin, and Jardiance but I’m not taking them.  (If asked why you’re not taking the meds at this point, respond with I know I’m supposed to take them, but I just don’t. For now, I just wanna give you my history and hear about my blood pressure and breathing)  Over The Counter (OTC): none  Herbs/supplements: none |
| Immunizations | - Tetanus - Flu - Hepatitis - Pneumovax - HPV - Other |
| Tobacco products:   - Cigarettes - Cigar - Pipe - Chew - E-cigarettes | - Never - Past - year started/year quit - Current   - Quantity   - # of years |
| Alcohol   - Beer - Wine - Liquor - Other | - Never - Past - year started/year quit - Current   - Quantity   - # of years |
| Drugs   - Weed - Cocaine - Heroin - Meth - IV - Inhalants - Other | - Never - Past - year started/year quit - Current   - Quantity   - # of years |
| Diet (describe) | Sandwich or salad for lunch, and a healthy dinner. |
| Exercise (describe) | Walks around home neighborhood. |
| List any other important social history or information important to this case | When asked about difficulty taking medication, SP shares a barrier they have faced. SP can elaborate by sharing stories from a prior negative experience |
| Family history | |
| Mother, father, siblings, grandparents, and other significant findings | Grandparents: Unknown  Mother: still alive (age = 30 years older than you), has hypertension  Father: still alive (age = 30 years older than you), has hypertension  No siblings  No children or pets |
|  |  |
| Physical Exam - List exam maneuvers expected for this case and any abnormal findings that SP will simulate. (tenderness, hyper-hypo reflex, rebound, weakness, etc.) | |
| PHYSICAL EXAM FINDINGS |  |
| 1. Written in layperson’s terms | Heart: Normal heart sounds, no extra heart sounds.  Lungs: Clear lungs with good air movement. No wheezing. |
| 1. General appearance - affect, appearance, position of patient at opening (i.e., sitting, lying down, holding abdomen, etc.) | Calm, cooperative, well dressed. Sitting, Not in distress. |
| 1. Vital signs | Height and weight in chart – 5’ 10” and 190 lbs.  Vitals: BP: 176/99 mm Hg. Students can obtain heart and respiratory rate if they would like practice. |
| 1. Specific findings and affect | Normal affect. No additional findings. |
| 1. Response to certain physical movements | N/A |
|  |  |
| DIAGNOSIS AND DIFFERENTIAL | N/A |
| Diagnosis with support from positive and negative history and PE findings | N/A |
| Differential with support from positive and negative history and PE findings | N/A |
|  |  |
| MANAGEMENT OR DIAGNOSTIC PLAN | Student should end the encounter by offering support/resources for the patient’s barrier and reemphasizing a desire to help the patient achieve their goals |
|  |  |
| PROFESSIONALISM ISSUES OR CHALLENGES | Navigating conflicting biases/distrust |

*For this encounter, Standardized Patients (SPs) are selected to participate if they have in real life experienced a barrier to their own healthcare that they are willing to act out in this simulated encounter. Each SP will adjust their portrayed religious/spiritual background, sex, sexual orientation, gender expression, financial situation, and insurance status to reflect their lived experience. Specific barriers that can be used for this encounter include but are not limited to: cost of care, insurance coverage, lack of transportation, inflexible work schedule, distrust in the healthcare system (prior negative experiences with the system/providers), and language/cultural barriers.

| SETTING: outpatient, in patient, ED, home, nursing home, rehab, group, etc. | Outpatient |
| --- | --- |
| PATIENT PROFILE: Information about the “patient” that helps select an SP and helps the learner get an understanding of them as a person. SP will know more information about the patient than learner will ever ask but allows SP to portray a fully developed patient personality. If none of the items below are particulars for the case, please write “all may be used.” | |
| Case | Difficulty Breathing |
| Age range | Age is same as SP’s real age |
| Religious/spiritual background | All may be used (should match SP’s barrier if applicable*) |
| Sex (e.g., male, female, intersex, transwoman, transman) | All may be used (should match SP’s barrier if applicable*) |
| Sexual orientation (e.g., heterosexual, lesbian, gay, bisexual, pansexual, queer, asexual) | All may be used (should match SP’s barrier if applicable*) |
| Gender expression (e.g., man, woman, genderqueer) | All may be used (should match SP’s barrier if applicable*) |
| Race and ethnicity (e.g., to promote educational diversity, we use a diverse pool of SPs.) | To promote educational diversity, we use a diverse pool of SPs |
| Physical description (e.g., BMI, height range) | 5’10” and 190 lbs. (May not match SP) |
| Physical limitations | None |
| Patient appearance (e.g., disheveled, hospital gown, business casual, casual) | Casual |
| Moulage + location (e.g., none, bruises, scars, body piercing, tattoos) | None |
| Affect (e.g., pleasant, cooperative) | Patient should be tired and slightly uncomfortable/standoffish at first due to prior dissatisfaction with the healthcare system/previous providers |
| Family group (e.g., who is family, who they live with) | Lives with college roommate. No siblings, children, or pets. |
| Education | Completed high school. Currently in college. |
| Level of health literacy | Average |
| Employment, if any - present and past, noting any current stresses | Works at campus bookstore |
| Home/homeless - type of dwelling, number of stories, owned or rented | Living in rented apartment |
| Financial situation - any current stresses | All may be used (should match SP’s barrier if applicable*) |
| Insurance status (e.g., un/under/insured, public/private, HMO/PPO) | All may be used (should match SP’s barrier if applicable*) |
| Habits (i.e., diet, exercise, caffeine, smoking, alcohol, drugs) | None |
| Activities (i.e., hobbies, sports, clubs, friends) | SP answers with their real life hobbies |
| Typical day - what is the usual daily routine | Classes, work, homework, hobbies/spending time with friends |

| CASE INFORMATION | Difficulty Breathing |
| --- | --- |
| Chief Concern: What the patient will say when greeted by the student. The patient’s primary reason for seeking medical care often stated in their own words. | “Hard time breathing” |
| Additional Concerns: Other, if any, concerns the patient has today (i.e., symptoms, requests, expectations, etc.) that will become part of set agenda. | None |
| THE PATIENT’S STORY: The SP will be asked to tell their symptom story and the personal and emotion impact for each of their concerns. You will want to write this in the patient’s voice. The symptom story should be able to answer this question: “Tell me more about [chief concern/additional concern], starting at the beginning and bringing me up to now.”  The personal context should be able to answer questions concerning the broader personal/psychosocial context of symptoms, especially the patient’s beliefs/attributions.  The emotional context should be able to ask how are you doing with this, how does this make you feel, how has this affected you emotionally? IMPACT: How has this affected your life? How has this been for your family? | Story: I’ve felt short of breath all week. I’ve felt like this before but it’s never lasted this long before. Sometimes, I’ll just be laughing and start coughing or wheezing.  Context: (Patient should be tired and slightly uncomfortable/standoffish at first due to prior dissatisfaction with the healthcare system/previous providers. Patient should give little to no detail about personal barriers until after the social history if the student has made them feel comfortable and heard.)  Impact: It makes it hard to focus or concentrate on anything |
| HISTORY OF PRESENT ILLNESS: Although some of the HPI will be given in the patient’s symptom story, the learners will expand the story during the direct question section. Below, describe the detailed history, usually about the chief concern, which the student must develop in order to make a useful assessment of the problem: | |
| Onset (when; gradual or sudden) | I’ve had breathing issues since I was little (age 7) but it feels like it started getting worse about a week ago. |
| Setting (what was going on or where was patient when symptoms first noticed?) | Nothing has changed recently and no recent illness. |
| Duration (how long) | Hard to breathe all the time |
| Time relationships (frequency, constant or intermittent) | Constant |
| Location | No chest pain but chest feels tight all over (on both sides). |
| Radiation | None |
| Quality | Chest tightness all over.  Cough is dry. Not coughing anything up.  Hard to take deep breaths and hard to breathe out specifically. |
| Amount | Chest tightness is 4/10.  Cough is 6/10.  Coughs and wheezes about 1-2x/day. |
| Aggravated by what | Breathing gets worse when I’m walking/exercising and it gets worse at night. Also gets worse when walking outside in the cold. |
| Relieved by what | Nothing makes it feel better. |
| Associated with what | Some wheezing, dry cough. No fevers, chills, sore throat, nasal congestion.  If asked, has not had a blood sugar level checked recently. |
| Attitude (what does the patient think is the problem, and how do they feel about it) | I’m getting worried that something’s really wrong with my lungs |
| Overall course | Progressively worsening over the past week |
| REVIEW OF SYSTEMS: Significant positives and negatives | |
|  | Positive for shortness of breath, wheezing, dry cough |
|  | Positive for polydipsia (thirsty all the time) |
|  | Negative for chest pain, fever, congestion, polyuria, lightheadedness, nausea, vomiting |
| Past medical history | Childhood: Asthma  Adult: Type I Diabetes  If asked, no history of thyroid, cortisol, or aldosterone issues  Psychiatric: None |
| Medication allergies (name and reaction) | None |
| Environmental allergies (name and reaction) | None |
| Illnesses | None |
| Vaccinations | Up to date |
| Surgeries | None |
| Accidents/injuries/trauma | None |
| Hospitalization | None |
| Inclusive sexual and reproductive history | |
| Sexual practices  Sexual partners  Protection: Use of safer sex practices  Use of birth control if appropriate  Risk of intimate partner violence | Sexually active, 1 lifetime partner, uses contraceptives, no history of STIs, has experienced vaginal sex (answer can be “no intimate sexual experiences” if contradictory with religion)  No risk of intimate partner violence. |
| OB/GYN history  Age of onset of menses  Age of menopause  Number of pregnancies  Number of live births  Number of miscarriages  Number of abortions | No previous pregnancies or live births. Last menstrual period was 2 weeks ago. Periods are regular and monthly, with some cramps. |
| Medications  Prescription/dose/reason  Over the counter/dose/reason  Herbs/supplements/dose/reason  Other: | I’m taking insulin (Humalog) every day 15 minutes before meals for diabetes. I used to have an inhaler for asthma but I don’t have one right now.  (If asked why you’re not using an inhaler at this point, respond with “I know I’m supposed to use one, but I just don’t. For now, I just wanna give you my history and hear about my breathing”)  Over The Counter (OTC): none  Herbs/supplements: none |
| Immunizations | - Tetanus - Flu - Hepatitis - Pneumovax - HPV - Other |
| Tobacco products:   - Cigarettes - Cigar - Pipe - Chew - E-cigarettes | - Never - Past - year started/year quit - Current   - Quantity   - # of years |
| Alcohol   - Beer - Wine - Liquor - Other | - Never - Past - year started/year quit - Current   - Quantity   - # of years |
| Drugs   - Weed - Cocaine - Heroin - Meth - IV - Inhalants - Other | - Never - Past - year started/year quit - Current   - Quantity   - # of years |
| Diet (describe) | Sandwich or salad for lunch, and a healthy dinner. |
| Exercise (describe) | Walks around campus, occasionally goes to the campus recreation center |
| List any other important social history or information important to this case | When asked about difficulty taking medication, SP shares a barrier they have faced. SP can elaborate by sharing stories from a prior negative experience |
| Family history | |
| Mother, father, siblings, grandparents, and other significant findings | Grandparents: Unknown  Mother: still alive (age = 30 years older than SP), has asthma  Father: still alive (age = 30 years older than SP), has asthma  No siblings  No children or pets |
|  |  |
| Physical Exam - List exam maneuvers expected for this case and any abnormal findings that SP will simulate. (tenderness, hyper-hypo reflex, rebound, weakness, etc.) | |
| PHYSICAL EXAM FINDINGS |  |
| 1. Written in layperson’s terms | Heart: Normal heart sounds, no extra heart sounds.  Lungs: Clear lungs with wheezing all over both lungs. |
| 1. General appearance - affect, appearance, position of patient at opening (i.e., sitting, lying down, holding abdomen, etc.) | Calm, cooperative, well dressed. Sitting, Not in distress. |
| 1. Vital signs | Height and weight in chart – 5’ 10” and 190 lbs.  Vitals: BP: 116/79 mm Hg.  Students can obtain heart rate and respiratory rate if they would like practice. |
| 1. Specific findings and affect | Normal affect. No additional findings. |
| 1. Response to certain physical movements | N/A |
|  |  |
| DIAGNOSIS AND DIFFERENTIAL | N/A |
| Diagnosis with support from positive and negative history and PE findings | N/A |
| Differential with support from positive and negative history and PE findings | N/A |
|  |  |
| MANAGEMENT OR DIAGNOSTIC PLAN | Student should end the encounter by offering support/resources for the patient’s barrier and reemphasizing a desire to help the patient achieve their goals |
|  |  |
| PROFESSIONALISM ISSUES OR CHALLENGES | Navigating conflicting biases/distrust |

*For this encounter, Standardized Patients (SPs) are selected to participate if they have in real life experienced a barrier to their own healthcare that they are willing to act out in this simulated encounter. Each SP will adjust their portrayed religious/spiritual background, sex, sexual orientation, gender expression, financial situation, and insurance status to reflect their lived experience. Specific barriers that can be used for this encounter include but are not limited to: cost of care, insurance coverage, lack of transportation, inflexible work schedule, distrust in the healthcare system (prior negative experiences with the system/providers), and language/cultural barriers.
