# Supplementary material for: Barriers to Care Encounter: A Model That Empowers Underserved Populations and Promotes Cross-Cultural Preparedness in Medical Students
Source: MedEdPORTAL. 2026 Jun 11;22:11608. doi: 10.15766/mep_2374-8265.11608 (PMC13253653; doi:10.15766/mep_2374-8265.11608)
Supplement: Supplementary file 1 — SP Case.docxLecture and Prebrief.pptxStudent Preencounter Instructions.docxStudent Guide for Gathering a History.docxPreencounter Survey.docxCommunication Skills Checklist.docxDebrief Discussion Questions.docxPostencounter Debrief Presentation.pptxPostencounter Survey.docxRecruitment Flyer.docxCase Overview and SP Training.docx [file mep_2374-8265.11608-s001.zip › B. Lecture and Prebrief.pptx]

## Slide 1
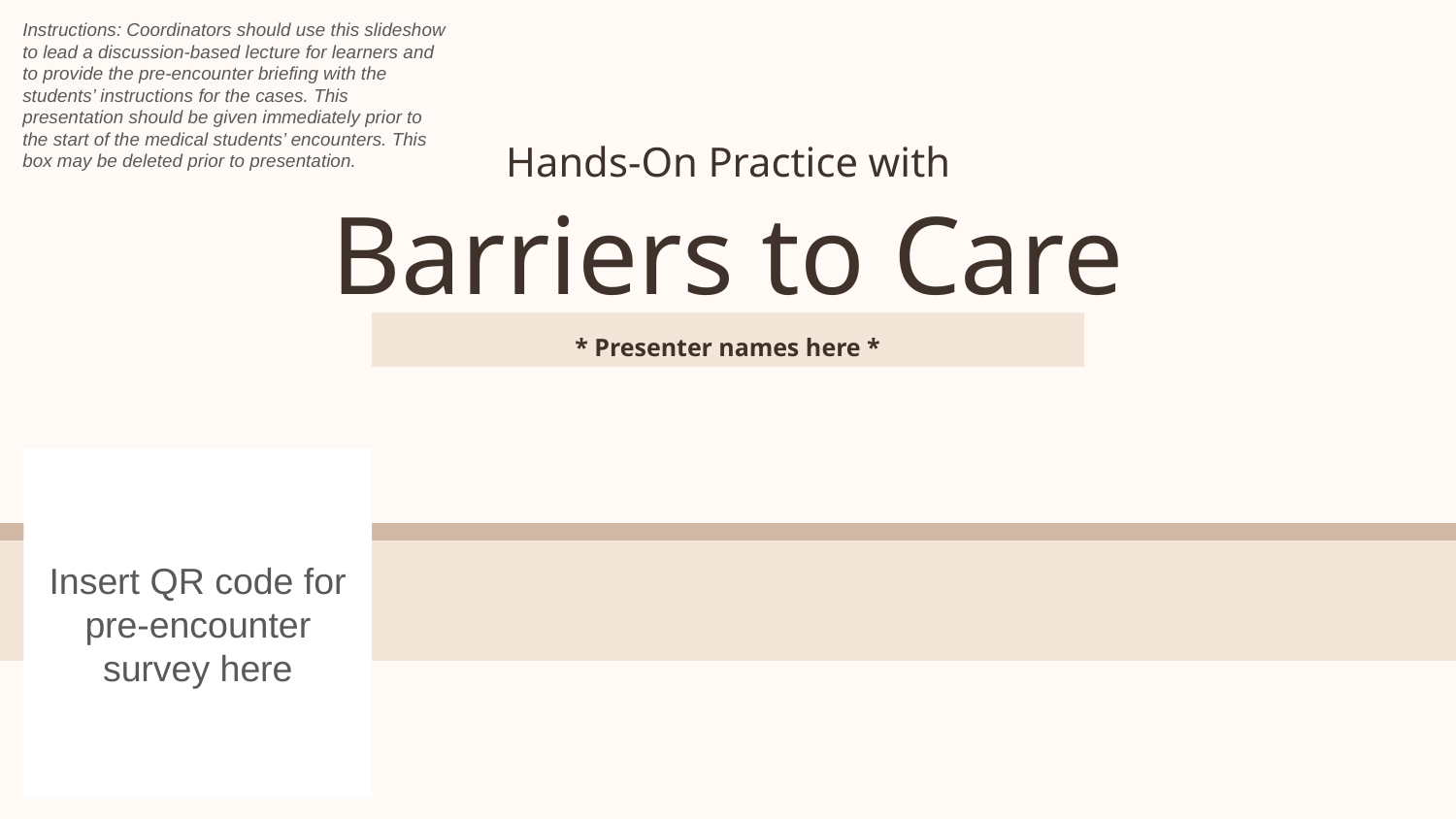

Instructions: Coordinators should use this slideshow to lead a discussion-based lecture for learners and to provide the pre-encounter briefing with the students’ instructions for the cases. This presentation should be given immediately prior to the start of the medical students’ encounters. This box may be deleted prior to presentation.
Hands-On Practice with
Barriers to Care
* Presenter names here *
Insert QR code for pre-encounter survey here

## Slide 2
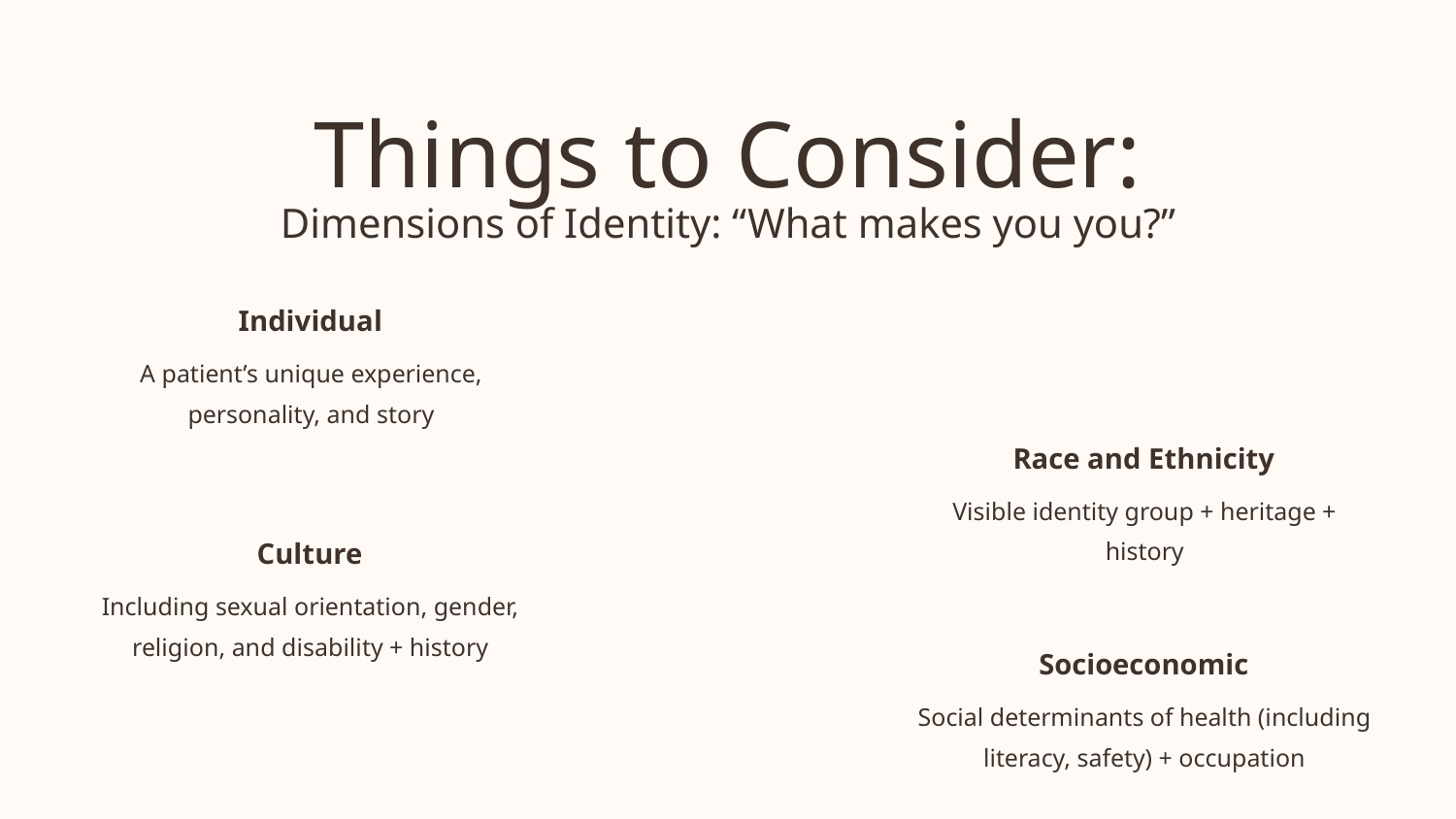

Things to Consider:
Dimensions of Identity: “What makes you you?”
Individual
A patient’s unique experience, personality, and story
Race and Ethnicity
Visible identity group + heritage + history
Culture
Including sexual orientation, gender, religion, and disability + history
Socioeconomic
Social determinants of health (including literacy, safety) + occupation

## Slide 3
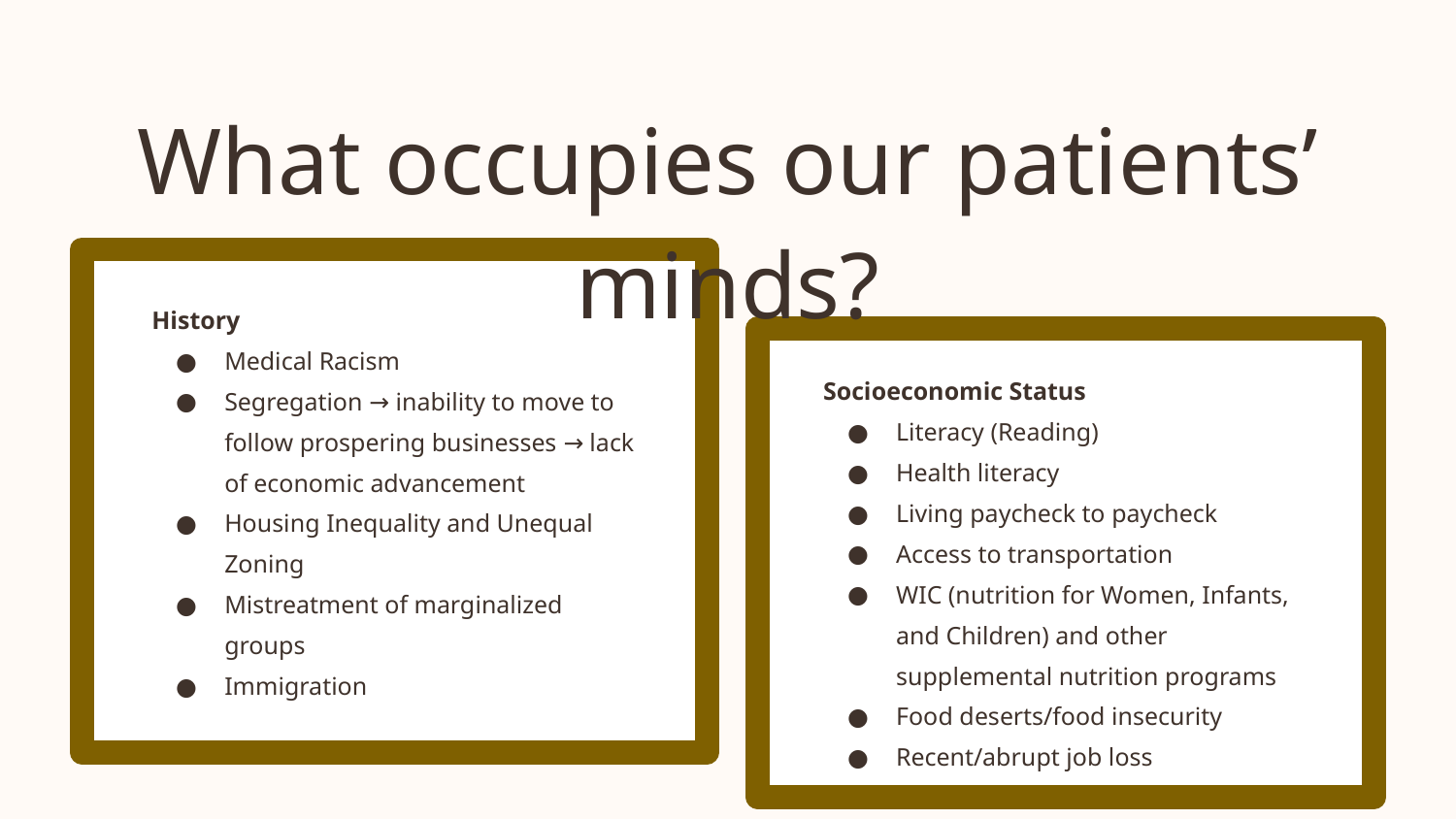

What occupies our patients’ minds?
History
Medical Racism
Segregation → inability to move to follow prospering businesses → lack of economic advancement
Housing Inequality and Unequal Zoning
Mistreatment of marginalized groups
Immigration
Socioeconomic Status
Literacy (Reading)
Health literacy
Living paycheck to paycheck
Access to transportation
WIC (nutrition for Women, Infants, and Children) and other supplemental nutrition programs
Food deserts/food insecurity
Recent/abrupt job loss

## Slide 4
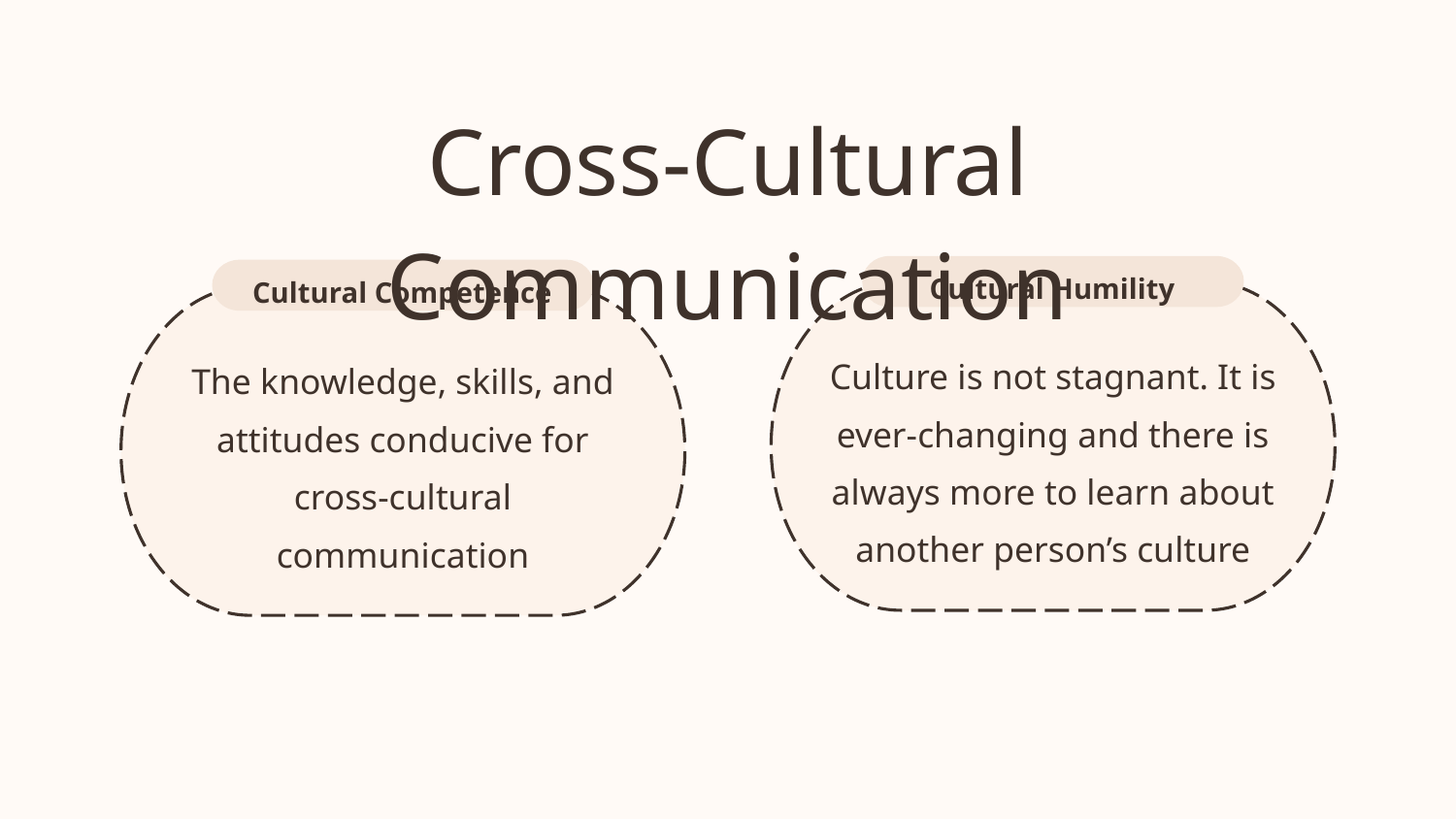

Cross-Cultural Communication
Cultural Humility
Cultural Competence
Culture is not stagnant. It is ever-changing and there is always more to learn about another person’s culture
The knowledge, skills, and attitudes conducive for cross-cultural communication

## Slide 5
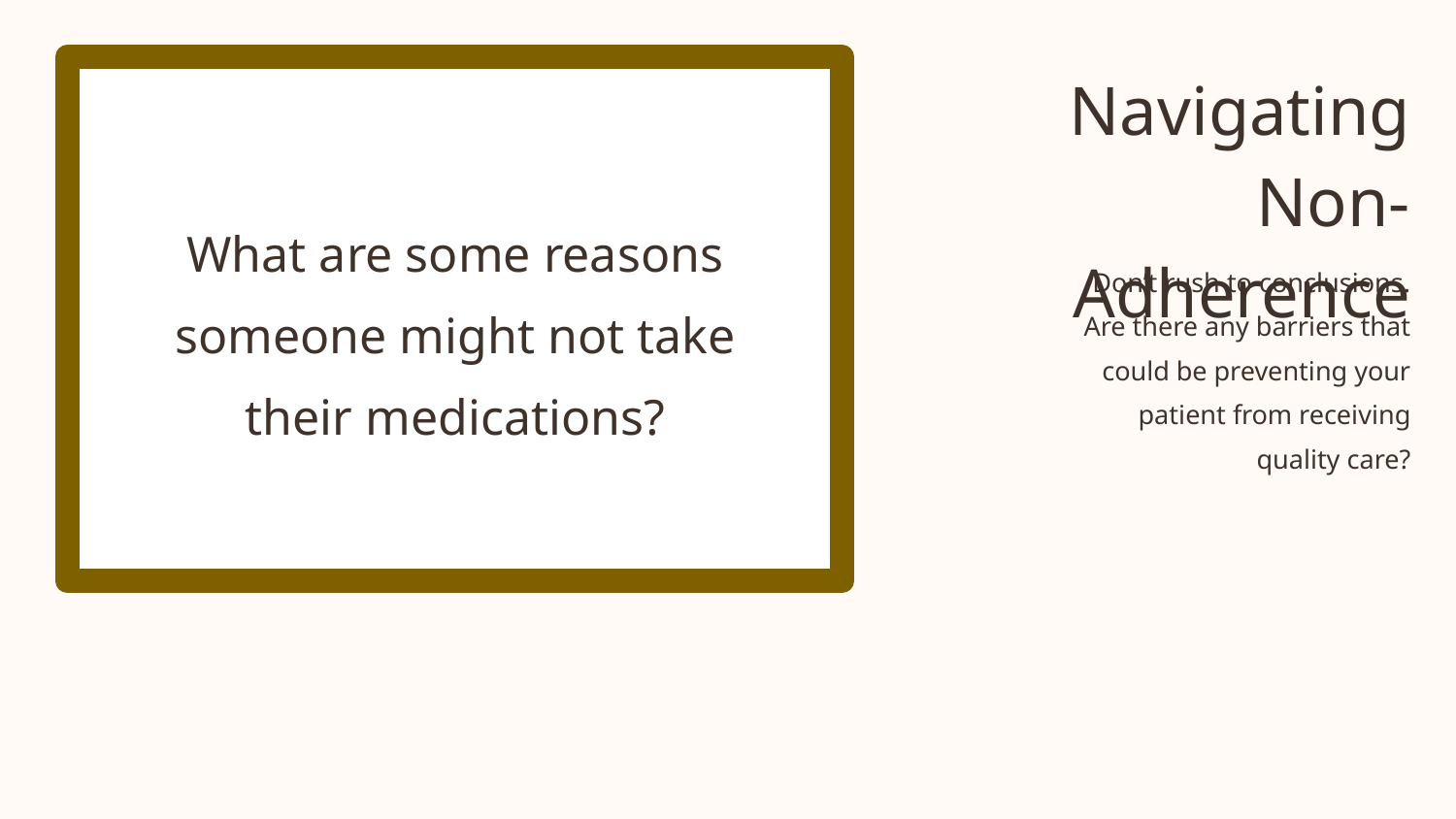

Navigating Non-Adherence
What are some reasons someone might not take their medications?
Don’t rush to conclusions.
Are there any barriers that could be preventing your patient from receiving quality care?

## Slide 6
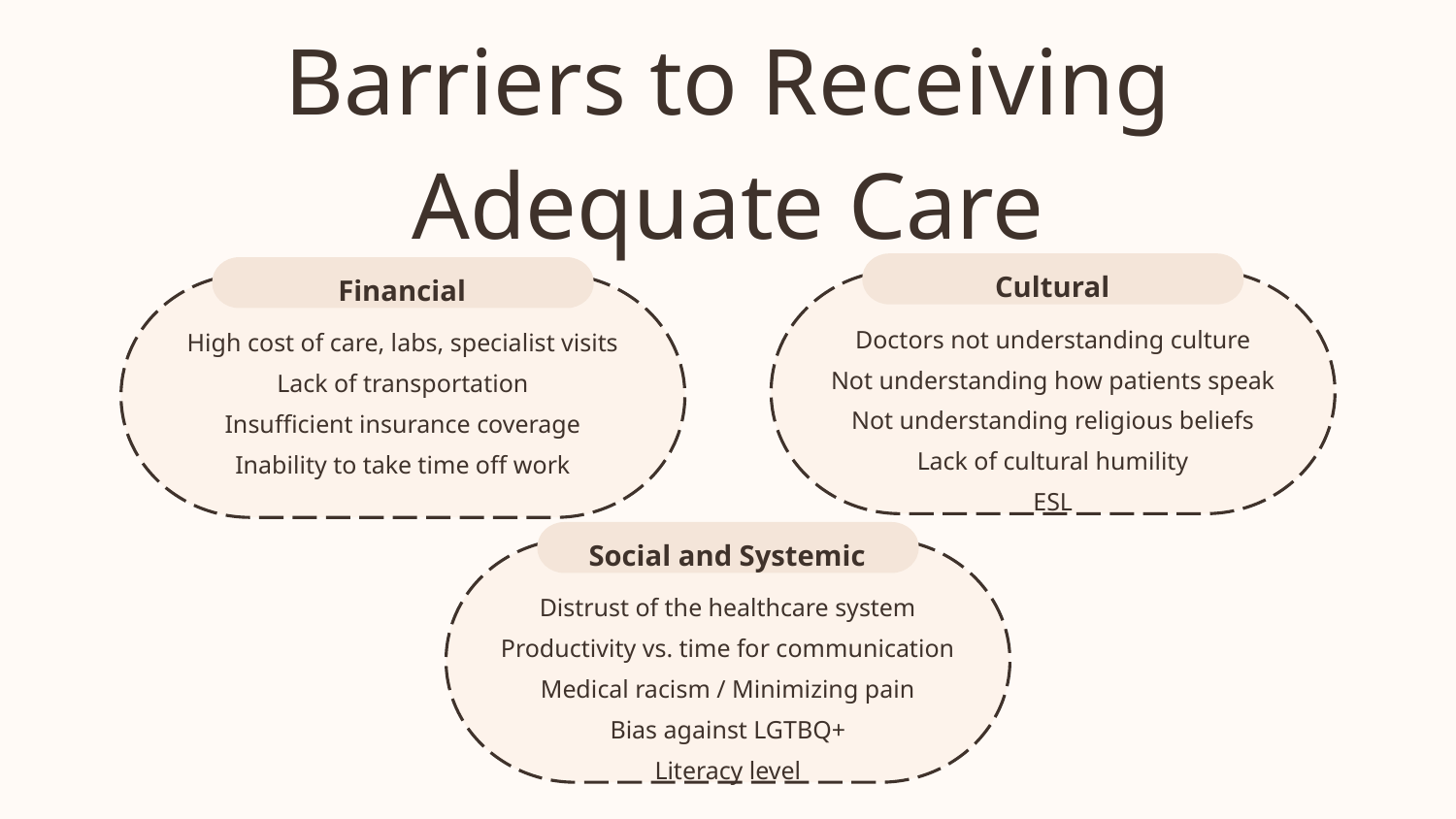

Barriers to Receiving Adequate Care
Cultural
Financial
Doctors not understanding culture
Not understanding how patients speak
Not understanding religious beliefs
Lack of cultural humility
ESL
High cost of care, labs, specialist visits
Lack of transportation
Insufficient insurance coverage
Inability to take time off work
Social and Systemic
Distrust of the healthcare system
Productivity vs. time for communication
Medical racism / Minimizing pain
Bias against LGTBQ+
Literacy level

## Slide 7
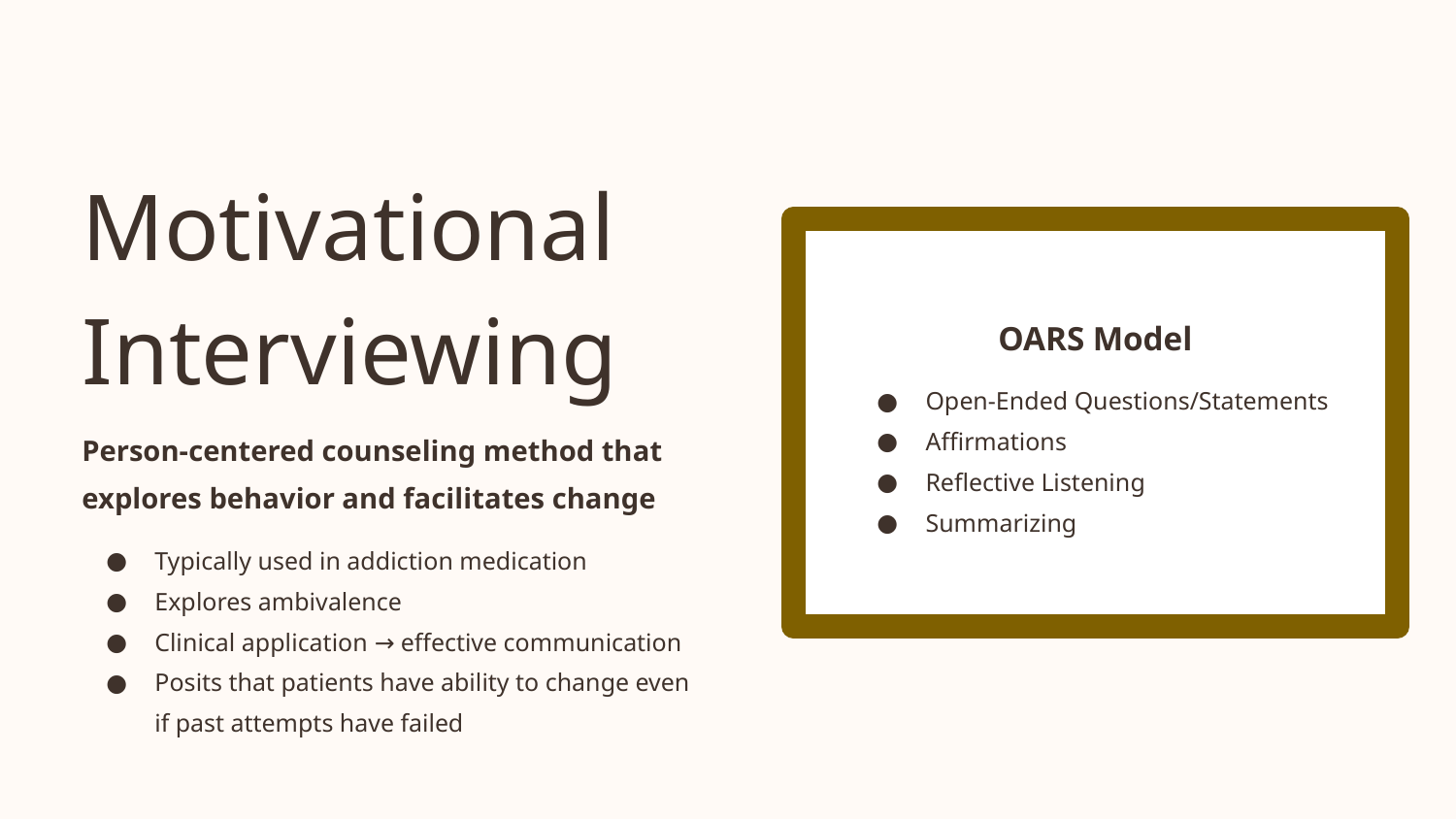

Motivational Interviewing
OARS Model
Open-Ended Questions/Statements
Affirmations
Reflective Listening
Summarizing
Person-centered counseling method that explores behavior and facilitates change
Typically used in addiction medication
Explores ambivalence
Clinical application → effective communication
Posits that patients have ability to change even if past attempts have failed

## Slide 8
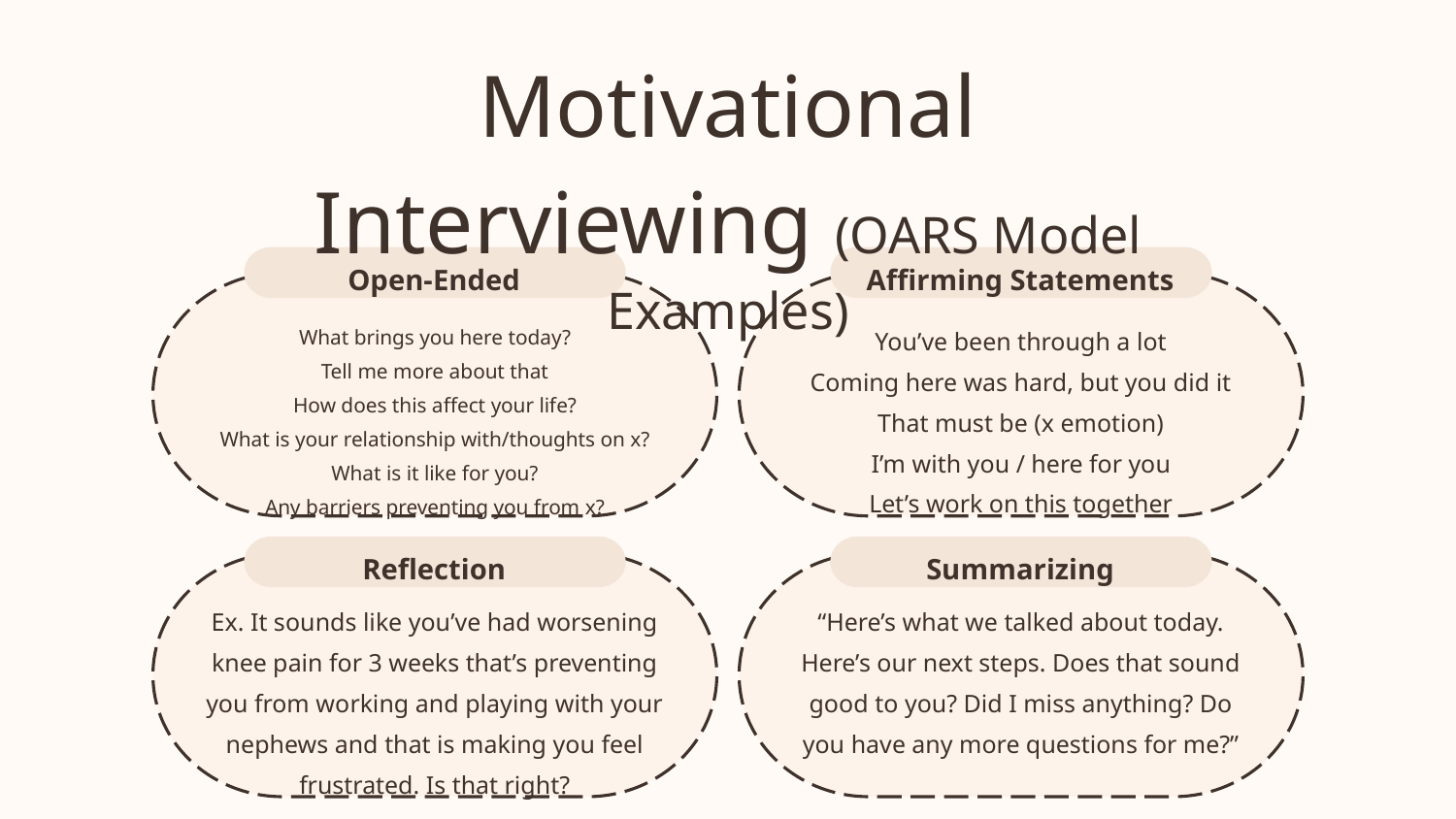

Motivational Interviewing (OARS Model Examples)
Open-Ended
Affirming Statements
What brings you here today?
Tell me more about that
How does this affect your life?
What is your relationship with/thoughts on x?
What is it like for you?
Any barriers preventing you from x?
You’ve been through a lot
Coming here was hard, but you did it
That must be (x emotion)
I’m with you / here for you
Let’s work on this together
Reflection
Summarizing
Ex. It sounds like you’ve had worsening knee pain for 3 weeks that’s preventing you from working and playing with your nephews and that is making you feel frustrated. Is that right?
“Here’s what we talked about today. Here’s our next steps. Does that sound good to you? Did I miss anything? Do you have any more questions for me?”

## Slide 9
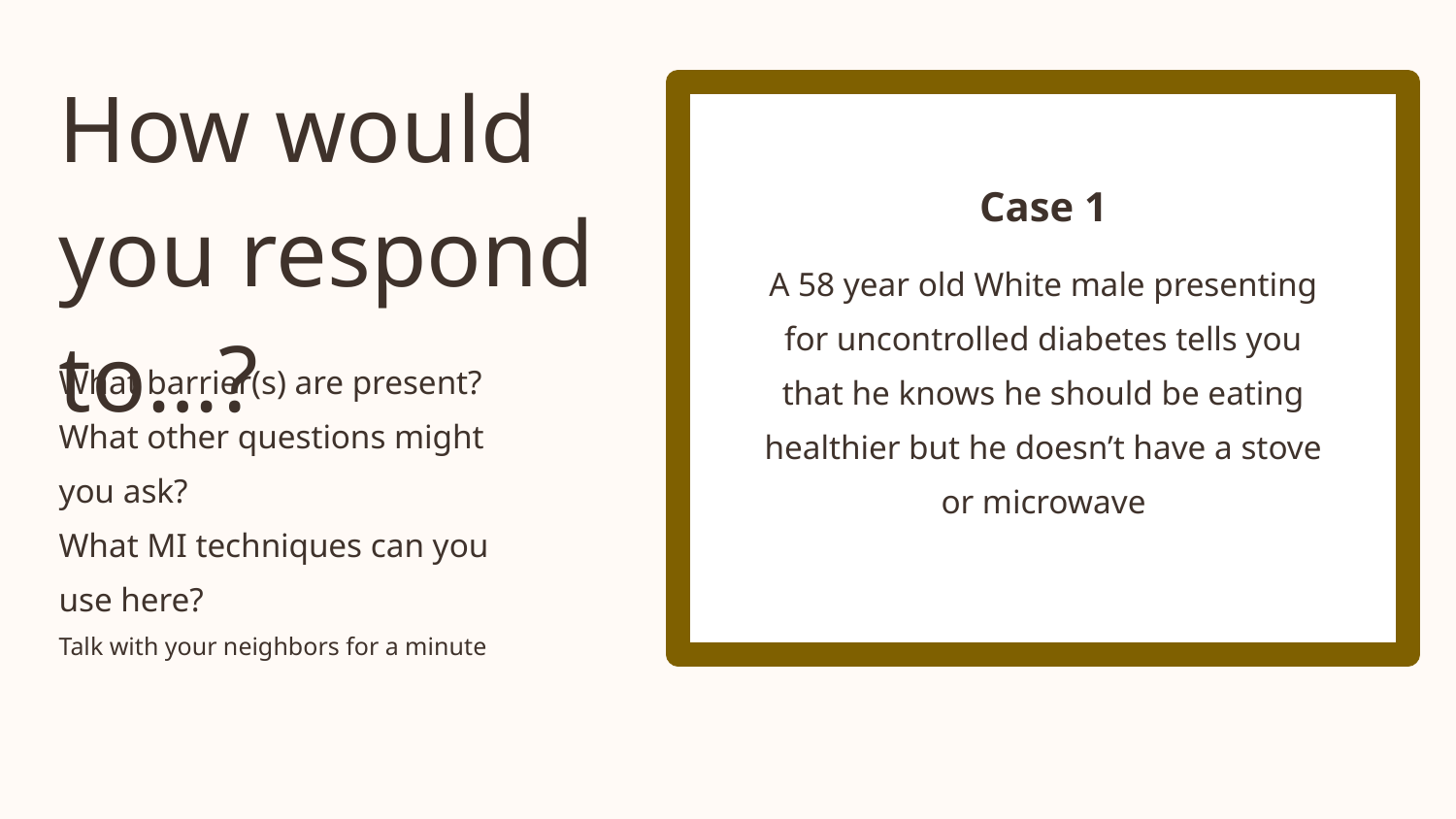

How would you respond to…?
Case 1
A 58 year old White male presenting for uncontrolled diabetes tells you that he knows he should be eating healthier but he doesn’t have a stove or microwave
What barrier(s) are present?
What other questions might you ask?
What MI techniques can you use here?
Talk with your neighbors for a minute

## Slide 10
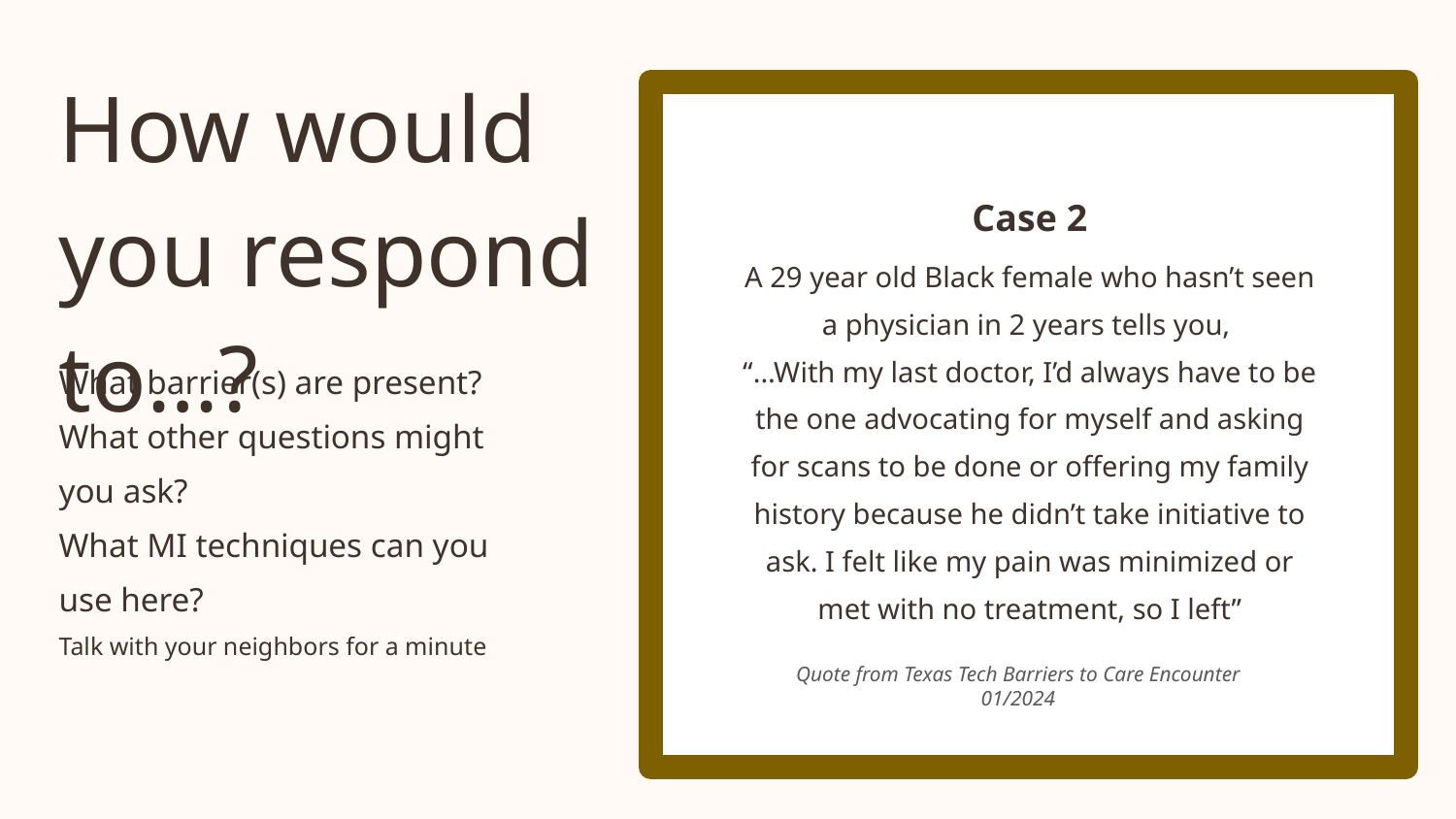

How would you respond to…?
Case 2
A 29 year old Black female who hasn’t seen a physician in 2 years tells you,
“...With my last doctor, I’d always have to be the one advocating for myself and asking for scans to be done or offering my family history because he didn’t take initiative to ask. I felt like my pain was minimized or met with no treatment, so I left”
What barrier(s) are present?
What other questions might you ask?
What MI techniques can you use here?
Talk with your neighbors for a minute
Quote from Texas Tech Barriers to Care Encounter 01/2024

## Slide 11
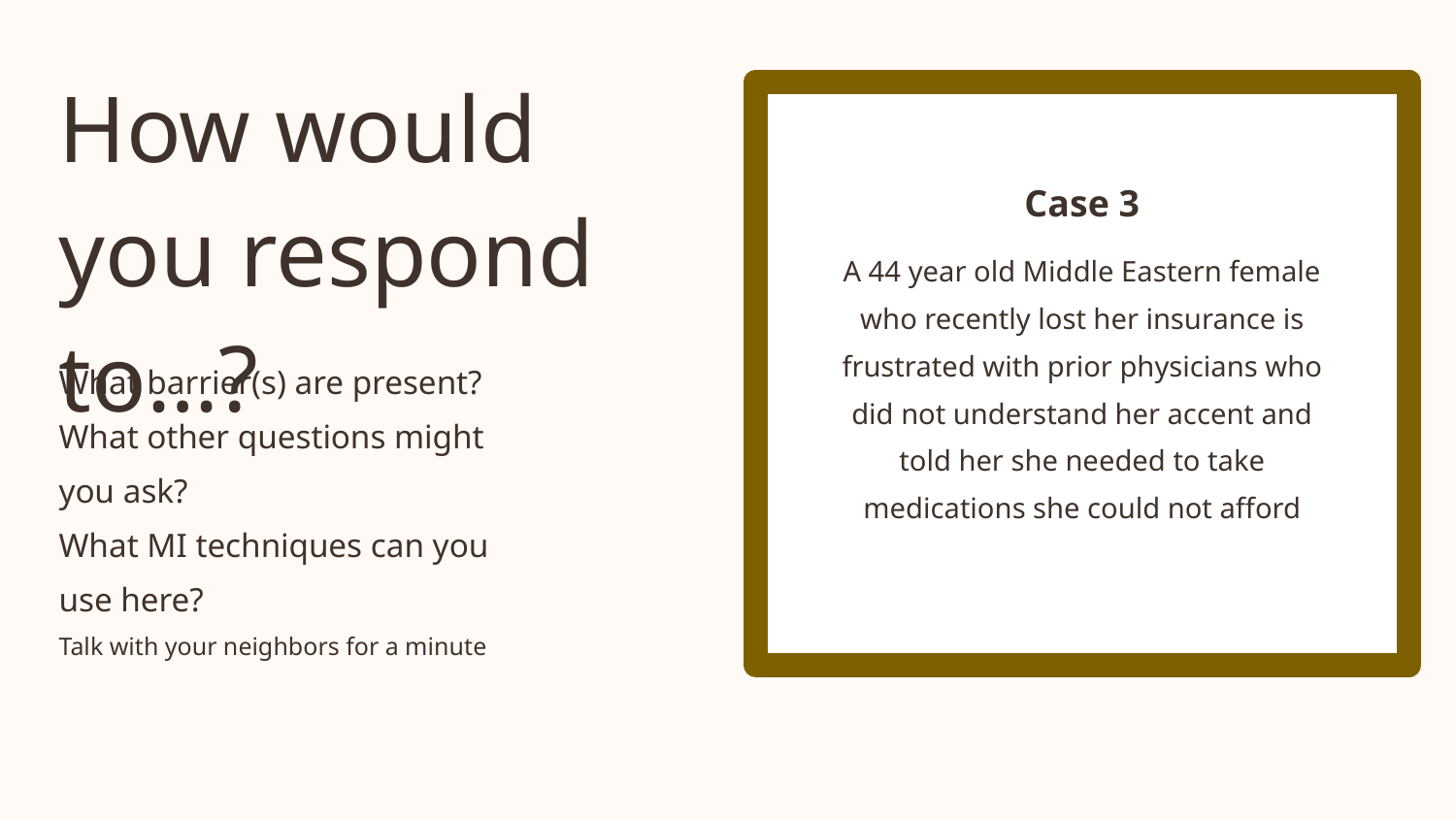

How would you respond to…?
Case 3
A 44 year old Middle Eastern female who recently lost her insurance is frustrated with prior physicians who did not understand her accent and told her she needed to take medications she could not afford
What barrier(s) are present?
What other questions might you ask?
What MI techniques can you use here?
Talk with your neighbors for a minute

## Slide 12
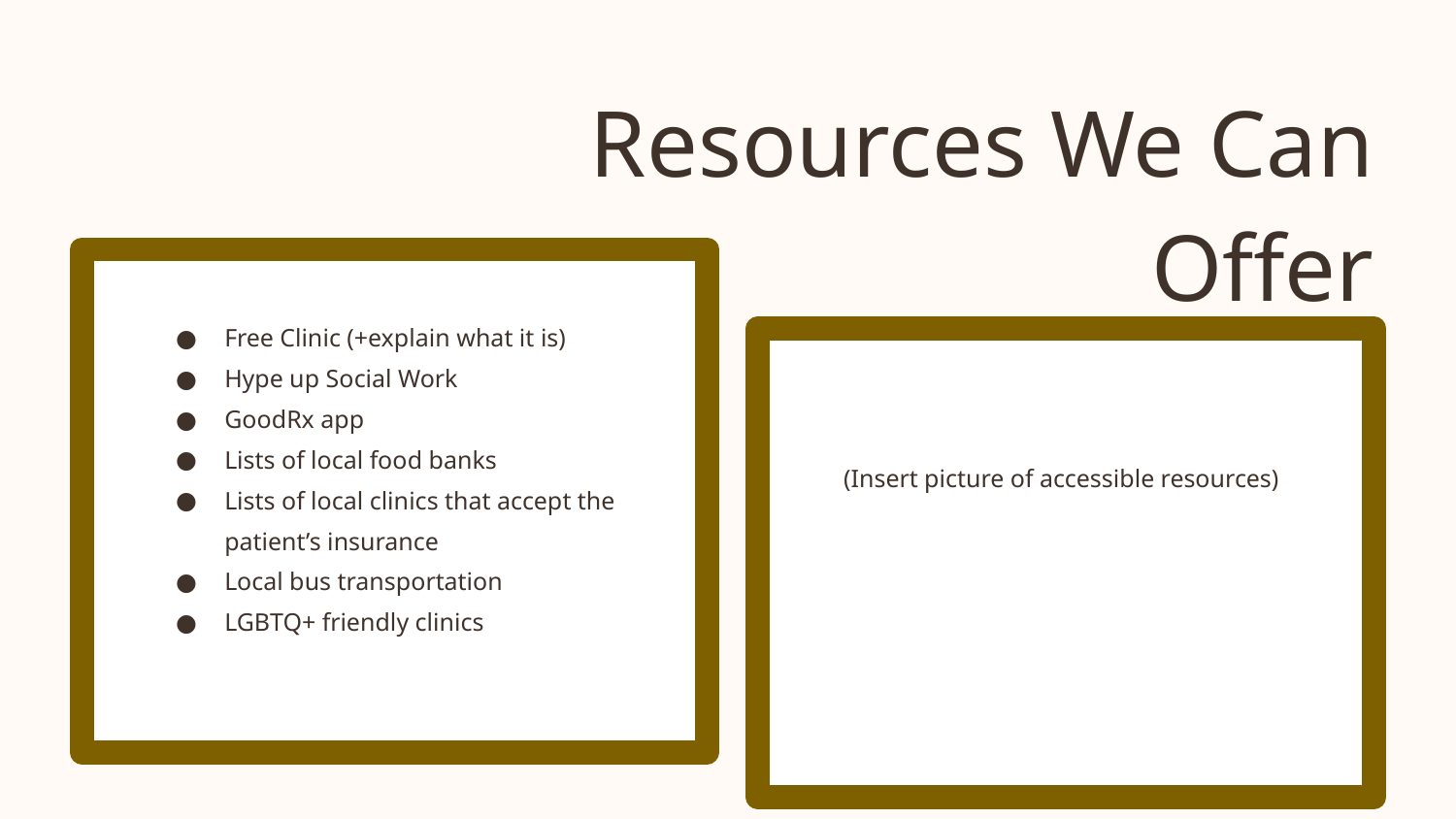

Resources We Can Offer
Free Clinic (+explain what it is)
Hype up Social Work
GoodRx app
Lists of local food banks
Lists of local clinics that accept the patient’s insurance
Local bus transportation
LGBTQ+ friendly clinics
(Insert picture of accessible resources)

## Slide 13
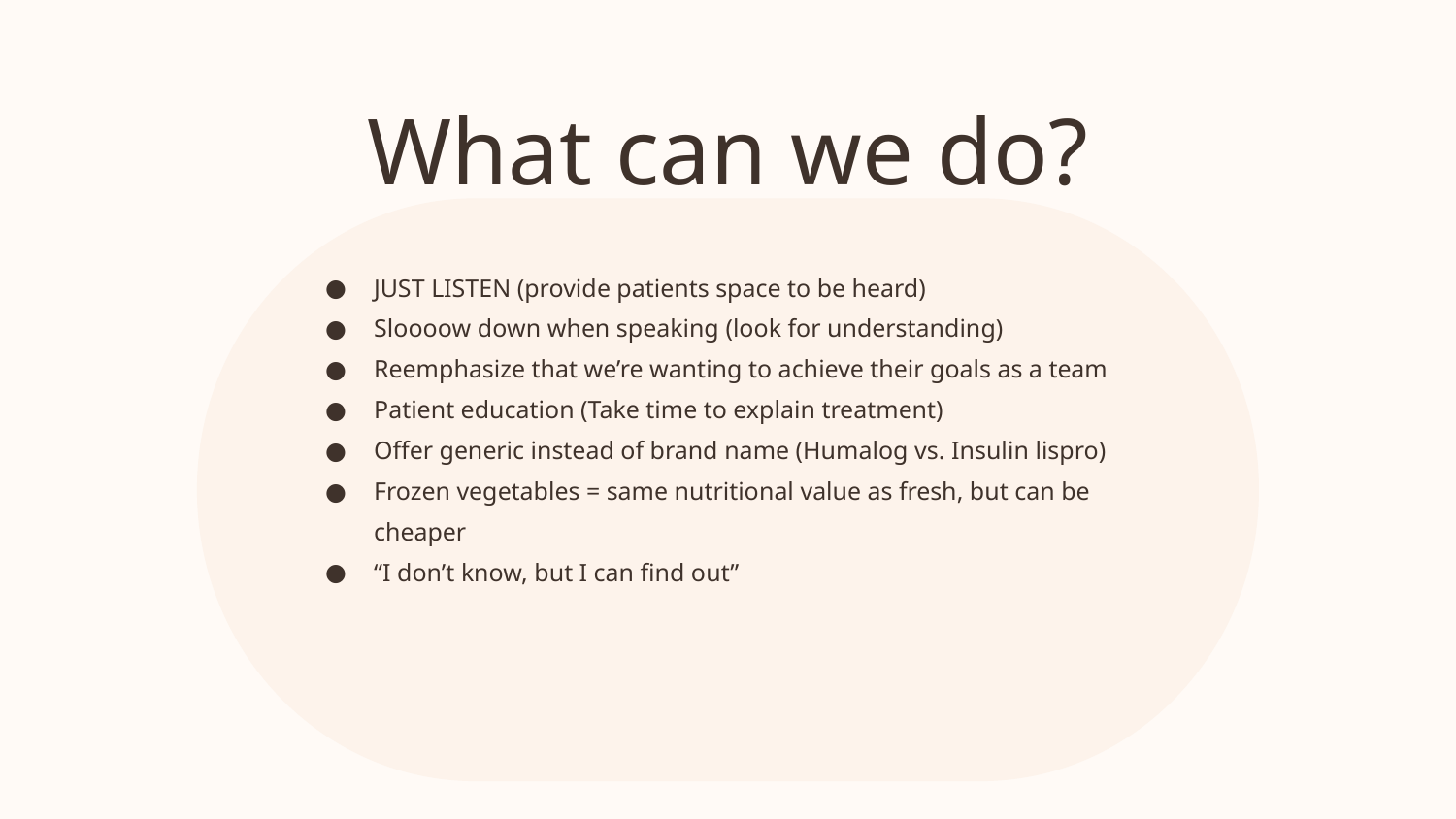

What can we do?
JUST LISTEN (provide patients space to be heard)
Sloooow down when speaking (look for understanding)
Reemphasize that we’re wanting to achieve their goals as a team
Patient education (Take time to explain treatment)
Offer generic instead of brand name (Humalog vs. Insulin lispro)
Frozen vegetables = same nutritional value as fresh, but can be cheaper
“I don’t know, but I can find out”

## Slide 14
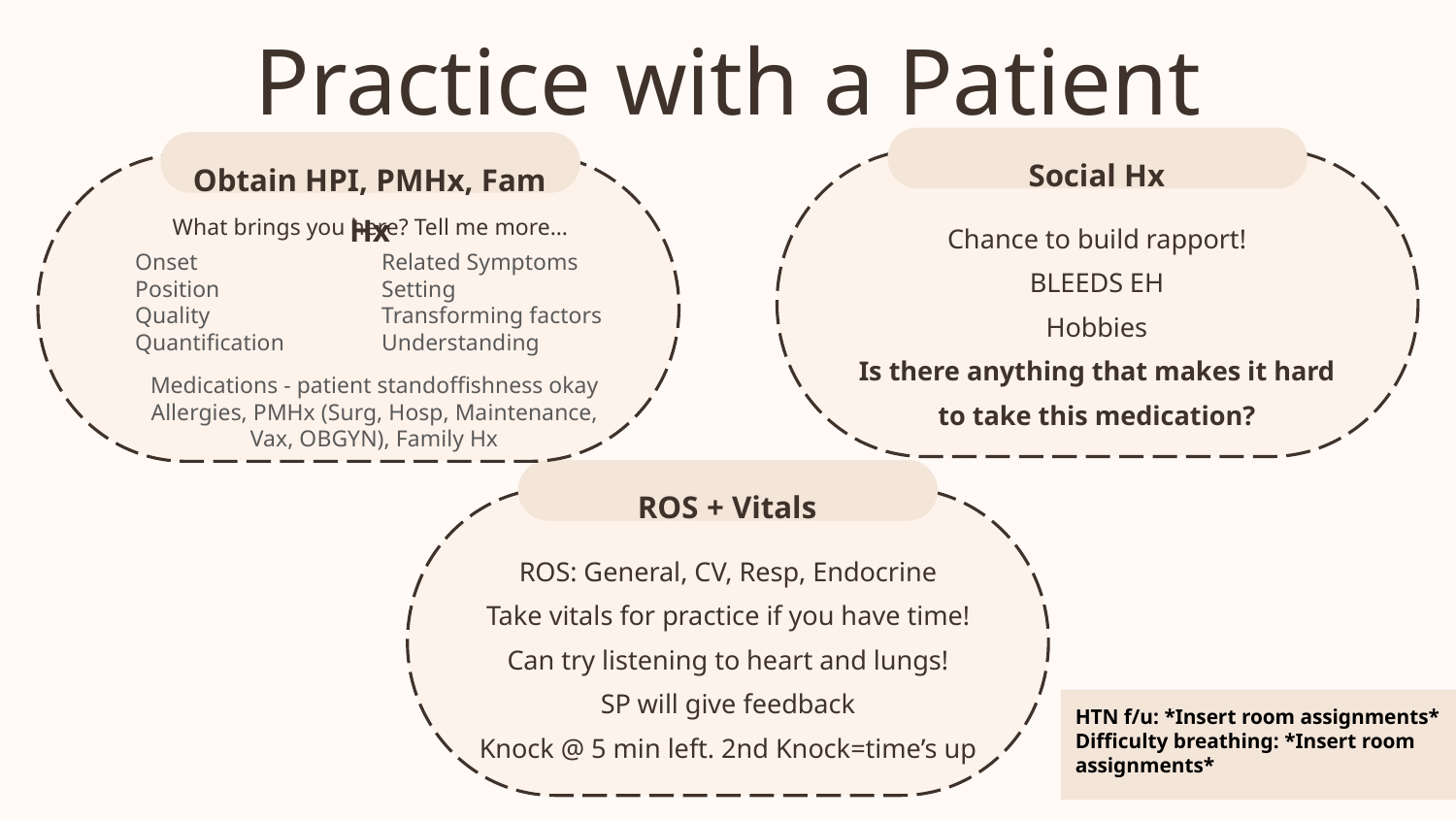

Practice with a Patient
Social Hx
Obtain HPI, PMHx, Fam Hx
What brings you here? Tell me more…
Chance to build rapport!
BLEEDS EH
Hobbies
Is there anything that makes it hard to take this medication?
Onset
Position
Quality
Quantification
Related Symptoms
Setting
Transforming factors
Understanding
Medications - patient standoffishness okay
Allergies, PMHx (Surg, Hosp, Maintenance, Vax, OBGYN), Family Hx
ROS + Vitals
ROS: General, CV, Resp, Endocrine
Take vitals for practice if you have time!
Can try listening to heart and lungs!
SP will give feedback
Knock @ 5 min left. 2nd Knock=time’s up
HTN f/u: *Insert room assignments*
Difficulty breathing: *Insert room assignments*
