# Supplementary material for: Barriers to Care Encounter: A Model That Empowers Underserved Populations and Promotes Cross-Cultural Preparedness in Medical Students
Source: MedEdPORTAL. 2026 Jun 11;22:11608. doi: 10.15766/mep_2374-8265.11608 (PMC13253653; doi:10.15766/mep_2374-8265.11608)
Supplement: Supplementary file 1 — SP Case.docxLecture and Prebrief.pptxStudent Preencounter Instructions.docxStudent Guide for Gathering a History.docxPreencounter Survey.docxCommunication Skills Checklist.docxDebrief Discussion Questions.docxPostencounter Debrief Presentation.pptxPostencounter Survey.docxRecruitment Flyer.docxCase Overview and SP Training.docx [file mep_2374-8265.11608-s001.zip › C. Student Preencounter Instructions.docx]

## Student Pre-Encounter Instructions

Students will encounter one of the following cases:

| **Station Name:** | **“**Maya/Michael Willow**”** |
| --- | --- |
| **Purpose of Case:** | - Demonstrate patient-centered communication skills using Motivational Interviewing (OARS Model)  - Obtain full health history and targeted ROS  - Establish rapport by getting to know your patient and facilitating a values-based conversation  - Suggest a treatment plan |
| **Setting:** | Family Medicine Clinic |
| **Presenting Situation:** | Hypertension follow up |
| **Vital Signs:** | 5’10” and 190 lbs. **BP: 176/99 mm Hg.** (May not match SP).  Obtain other vitals if you would like practice |
| **Allergies:** | Obtain |
| **Medications:** | Verify:  Metformin 500 mg PO BID for diabetes  Jardiance 10 mg PO daily for diabetes  Lisinopril 20 mg PO daily for hypertension |
| **ROS:** | Ask pertinent symptoms only (General, Cardiovascular, Respiratory, Endocrine) |
| **Physical Exam:** | Students may listen to Heart and Lungs if they would like |

| **Station Name:** | **“**Maya/Michael Willow**”** |
| --- | --- |
| **Purpose of Case:** | - Demonstrate patient-centered communication skills using Motivational Interviewing (OARS Model)  - Obtain full health history and targeted ROS  - Establish rapport by getting to know your patient and facilitating a values-based conversation  - Suggest a treatment plan |
| **Setting:** | Family Medicine Clinic |
| **Presenting Situation:** | Difficulty breathing |
| **Vital Signs:** | 5’10” and 190 lbs. **BP: 116/79 mm Hg.** (May not match SP).  Obtain other vitals if you would like practice |
| **Allergies:** | Obtain |
| **Medications:** | Verify:  Humalog (insulin) 100 units/mL (max: 30 units/injection) injected,  ProAir HFA (Albuterol inhaler) 8.5 g of 90 mcg inhaled, as needed |
| **ROS:** | Ask pertinent symptoms only (General, Cardiovascular, Respiratory, Endocrine) |
| **Physical Exam:** | Students may listen to Heart and Lungs if they would like |

**INSTRUCTIONS TO STUDENT:**

**1.** **You will have 15 minutes to take a focused medical history.**

**2.** **The standardized patient will take about 5 minutes to provide verbal feedback.**

**3. You will have a debrief session afterwards.**

**OARS Model for Motivational Interviewing***

- Asks 3 **open** ended questions (p. 41 SAMHSA MI) only some applicable
  - What brings you in today?
  - Tell me more about
  - Tell me when…
  - What is that like for you?
  - What’s your relationship with/thoughts on x?
  - How is this affecting your life?
  - How would you like to go about this/how would you like things to change?
  - Barriers to care: “Are there any barriers that make it hard to get or take your medications?”
- **Affirming** (might be case by case) (framing statements with “you”)
  - Coming here was hard, but you did it
  - Going through that is hard, but you did it
  - (For follow-ups) you’ve been working hard
  - You’ve been through a lot
  - That must have been (x emotion)
  - We’re here for you
  - We’re with you
  - Let’s work on this together
- **Reflective** listening
  - Student restated/rephrased SP’s responses (Can focus on SP’s feelings)
- **Summarizing**
  - Student repeats big picture to SP before leaving. (Students select statements that had meaning to the SP such as SP’s values/desires.)
  - Student asks if they missed anything
  - Student asks SP if they have any more questions
