# Supplementary material for: Barriers to Care Encounter: A Model That Empowers Underserved Populations and Promotes Cross-Cultural Preparedness in Medical Students
Source: MedEdPORTAL. 2026 Jun 11;22:11608. doi: 10.15766/mep_2374-8265.11608 (PMC13253653; doi:10.15766/mep_2374-8265.11608)
Supplement: Supplementary file 1 — SP Case.docxLecture and Prebrief.pptxStudent Preencounter Instructions.docxStudent Guide for Gathering a History.docxPreencounter Survey.docxCommunication Skills Checklist.docxDebrief Discussion Questions.docxPostencounter Debrief Presentation.pptxPostencounter Survey.docxRecruitment Flyer.docxCase Overview and SP Training.docx [file mep_2374-8265.11608-s001.zip › D. Student Guide for Gathering a History.docx]

Student Guide for Gathering a Patient History

Time/Date of Interview:

Name: DOB/age: Gender/pronouns:

| **CHIEF COMPLAINT:**  **HISTORY OF PRESENT ILLNESS:**   - **O**nset - **P**osition/radiation - **Q**uality - **Q**uantification (frequency/duration/severity) - **R**elated symptoms - **S**etting - **T**ransforming factors - **U**nderstanding   **MEDICATIONS** (prescription/OTC/supplement)   \| Medication \| Dose/freq \| Route \| Purpose \| \| --- \| --- \| --- \| --- \| \|  \|  \|  \|  \| \|  \|  \|  \|  \| \|  \|  \|  \|  \|   **ALLERGIES**   \|  \| Substance \| Reaction \| \| --- \| --- \| --- \| \| DRUG \|  \|  \| \| FOOD \|  \|  \|   **MEDICAL HISTORY**   - Childhood - Adult - Surgeries/procedures - Hospitalizations - Injuries/accidents - Psychiatric history - Ob/gyn - Health maintenance - Immunizations | **FAMILY HISTORY:**  Grandparents   - Age - Conditions   Parents   - Age - Conditions   Siblings   - Age - Conditions   **SOCIAL HISTORY:**   - Birthplace - Living situation - Education - Employment - Sexual History   - Currently active?   - # lifetime partners   - Men/women/both - Diet: 24hr recall or typical diet - Exercise - Habits:  \| Substance \| Product \| Amount \| Age started \| Age quit \| \| --- \| --- \| --- \| --- \| --- \| \| Caffeine \|  \|  \|  \|  \| \| Tobacco (ppd x years) \|  \|  \|  \|  \| \| Alcohol \|  \|  \|  \|  \| \| Recreational drugs \|  \|  \|  \|  \|   REVIEW OF SYSTEMS (see next page) |
| --- | --- | --- | --- | --- | --- | --- | --- | --- | --- | --- | --- | --- | --- | --- | --- | --- | --- | --- | --- | --- | --- | --- | --- | --- | --- | --- | --- | --- | --- | --- | --- | --- | --- | --- | --- | --- | --- | --- | --- | --- | --- | --- | --- | --- | --- | --- | --- | --- | --- | --- | --- |

REVIEW OF SYSTEMS: “In the past 2 weeks have you experienced any of the following?”

| **GENERAL/CONSTITUTIONAL** |  | Abdominal pain |  |
| --- | --- | --- | --- |
| Fever/chills |  | Change in stool color/caliber |  |
| Weakness/fatigue |  | Black tarry stool (melena) |  |
| Weight loss/gain |  | Blood in stool (hematochezia) |  |
| **SKIN** |  | Hemorrhoids (painful?) |  |
| Change in hair/nails/moles |  | Diarrhea (color/consistency) |  |
| Loss of pigment |  | Constipation |  |
| Rashes/itching/hives |  | **URINARY** |  |
| **HEAD** |  | Blood in urine (hematuria) |  |
| Headache (type/frequency) |  | Increased frequency |  |
| Dizziness (fainting vs. vertigo) |  | Sense of urgency |  |
| Trauma |  | Get up at night to urinate (nocturia) |  |
| **EYES** |  | Pain with urinating (dysuria) |  |
| Corrective lenses |  | Difficulty starting stream (hesitancy) |  |
| Blurry vision |  | Incomplete emptying |  |
| Double vision (diplopia) |  | Incontinence (stress/urge/overflow) |  |
| Spots in your vision, floaters |  | **MALE GENITALIA** |  |
| Discharge |  | Penile discharge |  |
| Pain/redness |  | Testicular masses/pain |  |
| **EARS** |  | Libido/arousal problems |  |
| Hearing aids |  | Erectile dysfunction |  |
| Earaches |  | **FEMALE GENITALIA** |  |
| Discharge (color?) |  | Vaginal discharge |  |
| **MOUTH/THROAT** |  | Sores/lumps |  |
| Dentures |  | Libido problems |  |
| Bleeding gums |  | Pelvic pain (dyspareunia) |  |
| Hoarseness |  | Age of menarche (first period) |  |
| Sore throat |  | Frequency/duration of menses |  |
| Tooth pain |  | Dysmenorrhea/PMS |  |
| **NECK** |  | Bleeding between menses |  |
| Lumps/masses |  | Last menstrual period |  |
| Pain/stiffness |  | If menopause: hot flashes |  |
| Swollen glands |  | Post-menopausal bleeding |  |
| BREASTS |  | Vaginal dryness |  |
| Lumps/skin change |  | **MUSCULOSKELETAL** |  |
| Nipple discharge |  | Joint pain/stiffness |  |
| Pain |  | Muscle weakness |  |
| **PULMONARY** |  | Leg cramps |  |
| Cough (sputum?) |  | **NEUROLOGIC** |  |
| Coughing up blood (amount) |  | Fainting (syncope)/seizures |  |
| Shortness of breath (dyspnea) |  | Weakness/paralysis |  |
| Sharp pain with breathing (pleuritic pain) |  | Numbness/tingling |  |
| Loud snoring; stop breathing during sleep |  | Tremors |  |
| Wheezing |  | Poor balance |  |
| **CARDIOVASCULAR** |  | **HEME** |  |
| Chest pain/pressure |  | Easy bruising |  |
| Palpitations (too fast/too hard) |  | Easy bleeding |  |
| Shortness of breath with exertion |  | **ENDOCRINE** |  |
| Shortness of breath lying flat (orthopnea) |  | Polyuria, polydipsia, polyphagia |  |
| Shortness of breath waking up from sleep |  | Heat/cold intolerance |  |
| Swelling in legs (edema; how far up) |  | Change in glove/shoe size |  |
| Pain in legs with walking (claudication) |  | **PSYCHIATRIC** |  |
| Ulcers/sores, non-healing |  | Nervousness/anxiety |  |
| **GASTROINTESTINAL** |  | Sadness/depression |  |
| Change in appetite |  | Memory changes |  |
| Nausea/vomiting |  | Sleep changes |  |
| Trouble swallowing (dysphagia) |  | Suicide attempts |  |
| Pain with swallowing (odynophagia) |  | Hallucinations |  |
| Heartburn |  |  |  |
