# Supplementary material for: Barriers to Care Encounter: A Model That Empowers Underserved Populations and Promotes Cross-Cultural Preparedness in Medical Students
Source: MedEdPORTAL. 2026 Jun 11;22:11608. doi: 10.15766/mep_2374-8265.11608 (PMC13253653; doi:10.15766/mep_2374-8265.11608)
Supplement: Supplementary file 1 — SP Case.docxLecture and Prebrief.pptxStudent Preencounter Instructions.docxStudent Guide for Gathering a History.docxPreencounter Survey.docxCommunication Skills Checklist.docxDebrief Discussion Questions.docxPostencounter Debrief Presentation.pptxPostencounter Survey.docxRecruitment Flyer.docxCase Overview and SP Training.docx [file mep_2374-8265.11608-s001.zip › F. Communication Skills Checklist.docx]

## Communication Skills Checklist

| **Case: Hypertension/Asthma Management with Barriers to Care** | | **0**  **(No)** | **1**  **(Attempt)** | **2**  **(Yes)** |
| --- | --- | --- | --- | --- |
| 1. | The student knocks on the door and waits for you to say “Come in!” before walking into the room |  |  |  |
| 2. | The student closes the door behind them completely and then starts talking to you |  |  |  |
| 3. | The student introduces themselves and their role |  |  |  |
| 4. | The student confirms your legal name |  |  |  |
| 5. | The student asks you how you prefer to be addressed (Mr./Ms./Mrs., first name, etc.)/asks for your preferred name AFTER confirming your legal name |  |  |  |
| 6. | The student washes their hands or uses hand sanitizer (either at the beginning of the visit OR before listening to your heart and lungs) |  |  |  |
| 7. | The student shakes your hand |  |  |  |
| 8. | The student makes appropriate eye contact |  |  |  |
| 9. | The student asks about the chief complaint (main reason for coming to clinic) as an open-ended question* (Examples: What brings you in? Tell me why you’re here today.) |  |  |  |
| 10. | The student asked for more information by an open-ended question* (Example: Tell me more about that.) |  |  |  |
| 11. | The student asked or spoke about how this health issue is affecting your life |  |  |  |
| 12. | The student tried to connect with you and made you feel heard, appreciated, respected, or more comfortable)* |  |  |  |
| 13. | The student showed interest, concern, and respect |  |  |  |
| 14. | The student demonstrated good listening by repeating what you told them* |  |  |  |
| 15. | The student gave you a summary based on your concerns before leaving and asked if anything was missed* |  |  |  |
| 16. | The student allowed you to ask questions or add comments before leaving |  |  |  |
| 17. | The student asks about barriers to care (including costs of tests or medication, not having a car or a way to travel to appointments, or not having insurance, etc.)** |  |  |  |
| 18. | The student does not blame you for your circumstances (avoids shaming you for not taking medication(s))** |  |  |  |
| 19. | The student offers resources for help (free or low-cost clinics, local social work services, or low-cost pharmacy or medications, etc.)** |  |  |  |
| 20. | The student focuses on your goals and values to create a treatment plan for you** |  |  |  |

0-2 Scale: 0 - Was not done, 1 - was done partially, 2 - was done thoroughly

*These communication skills are specific to the Open-questions, Affirmations, Reflections, and Summarizing (OARS) Model for Motivational Interviewing.

**These communication skills are specific to conversations with barriers to healthcare.

References

1. “Communication Skills Checklist.” [class handout]. Development of Clinical Skills, School of Medicine, Texas Tech University Health Sciences Center, 2022. Lubbock, Texas.
