# Supplementary material for: Barriers to Care Encounter: A Model That Empowers Underserved Populations and Promotes Cross-Cultural Preparedness in Medical Students
Source: MedEdPORTAL. 2026 Jun 11;22:11608. doi: 10.15766/mep_2374-8265.11608 (PMC13253653; doi:10.15766/mep_2374-8265.11608)
Supplement: Supplementary file 1 — SP Case.docxLecture and Prebrief.pptxStudent Preencounter Instructions.docxStudent Guide for Gathering a History.docxPreencounter Survey.docxCommunication Skills Checklist.docxDebrief Discussion Questions.docxPostencounter Debrief Presentation.pptxPostencounter Survey.docxRecruitment Flyer.docxCase Overview and SP Training.docx [file mep_2374-8265.11608-s001.zip › G. Debrief Discussion Questions.docx]

Debrief Notes

Discussion Questions

1. What barriers did you notice in your patients?
2. Did anything surprise you?
3. Did you learn anything from your SP’s feedback?
4. Explain that all SPs’ barriers were true to their experiences (None of the barriers were made up). Many of the SPs were people of color or came from an underserved or historically disadvantaged background on purpose (with their permission)
   1. Oftentimes, barriers have historical roots. Even SES barriers can have intersectionality with a person’s other identity groups. Healthcare can be a luxury for people in these groups. Can you see how these (history and SES) affect each other?
   2. Can you think of what the historical roots of your SP’s barriers may have been?
   3. One of the goals of this encounter was to empower patients. Simulation activities without representation may limit our ability to highlight the structural factors that disempower patients. And that would further disempower these patients.
5. Cultural competence is defined as the knowledge, skills, and attitudes conducive for cross-cultural communication. This encounter focuses on communication skills (OARS model) but could you see how knowledge and attitudes plays into the patient-physician dynamic?
6. Was the OARS model helpful?
7. Ultimately, we’re only being “culturally competent” if our patients feel heard and understood. Was it helpful to hear your patient’s impression of you or how you came across to them?
   1. CPUP coordinator can explain the concept of cultural humility - lifelong learning, having the patient help YOU understand where they’re coming from, culture being ever-changing so it may not be possible to reach a point of “competence”

Please ensure that they take the Post-Survey!

**Examples of Barriers (for reference for question 1)**

- Distrust of the healthcare system
  - feeling unheard and/or unsafe
  - discrimination due to race/ethnicity, sexuality, disability status, religious beliefs and on the basis of other identity groups
- Doctor did not understand their cultural beliefs
- Doctor did not understand their religious beliefs
- Lack of transportation to appointments/specialists/labs
- Cost of care/lab tests
- Insufficient insurance coverage
- Literacy level
- Inability to take time off work

**OARS Model for Motivational Interviewing***

- Asks 3 **open** ended questions (p. 41 SAMHSA MI) only some applicable
  - What brings you in today?
  - Tell me more about…
  - Tell me when…
  - Tell me what it’s like when…
  - How is this affecting your life?
  - What do you want to do about____?
  - How would you like to go about this/how would you like things to change?
  - How would you like your life to be different a year from now?
  - What needs to happen?
  - Barriers to care: “Are there any barriers that make it hard to get or take your medications?”
- **Affirming** (might be case by case) (framing statements with “you”)
  - You took a big step in coming here today
  - Coming here was hard, but you did it
  - Going through that is hard, but you did it
  - (For follow-ups) you’ve been working hard
  - Things didn’t turn out how you wanted, but you tried really hard
  - I’m proud of you for coming in today
- **Reflective** listening (student restated/rephrased SP’s responses. Can focus SP’s feelings)
- **Summarizing** (student repeats big picture to SP before leaving. Students select statements that had meaning to the SP such as SP’s values/desires. Emphasizes “change talk” if applicable)
  - Asks if they missed anything
