# Supplementary material for: Barriers to Care Encounter: A Model That Empowers Underserved Populations and Promotes Cross-Cultural Preparedness in Medical Students
Source: MedEdPORTAL. 2026 Jun 11;22:11608. doi: 10.15766/mep_2374-8265.11608 (PMC13253653; doi:10.15766/mep_2374-8265.11608)
Supplement: Supplementary file 1 — SP Case.docxLecture and Prebrief.pptxStudent Preencounter Instructions.docxStudent Guide for Gathering a History.docxPreencounter Survey.docxCommunication Skills Checklist.docxDebrief Discussion Questions.docxPostencounter Debrief Presentation.pptxPostencounter Survey.docxRecruitment Flyer.docxCase Overview and SP Training.docx [file mep_2374-8265.11608-s001.zip › H. Postencounter Debrief Presentation.pptx]

## Slide 1
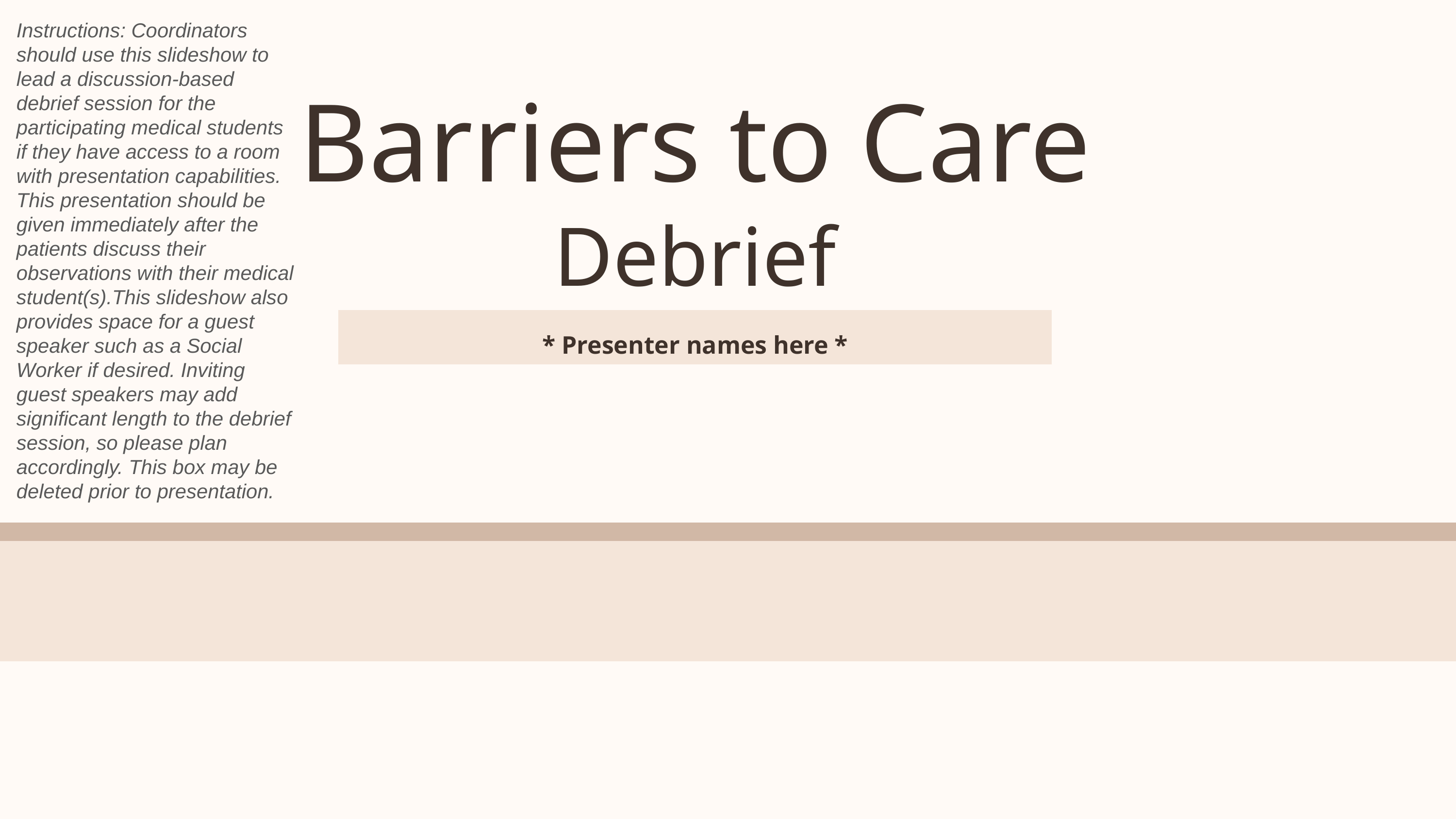

Instructions: Coordinators should use this slideshow to lead a discussion-based debrief session for the participating medical students if they have access to a room with presentation capabilities. This presentation should be given immediately after the patients discuss their observations with their medical student(s).This slideshow also provides space for a guest speaker such as a Social Worker if desired. Inviting guest speakers may add significant length to the debrief session, so please plan accordingly. This box may be deleted prior to presentation.
Barriers to Care Debrief
* Presenter names here *

## Slide 2
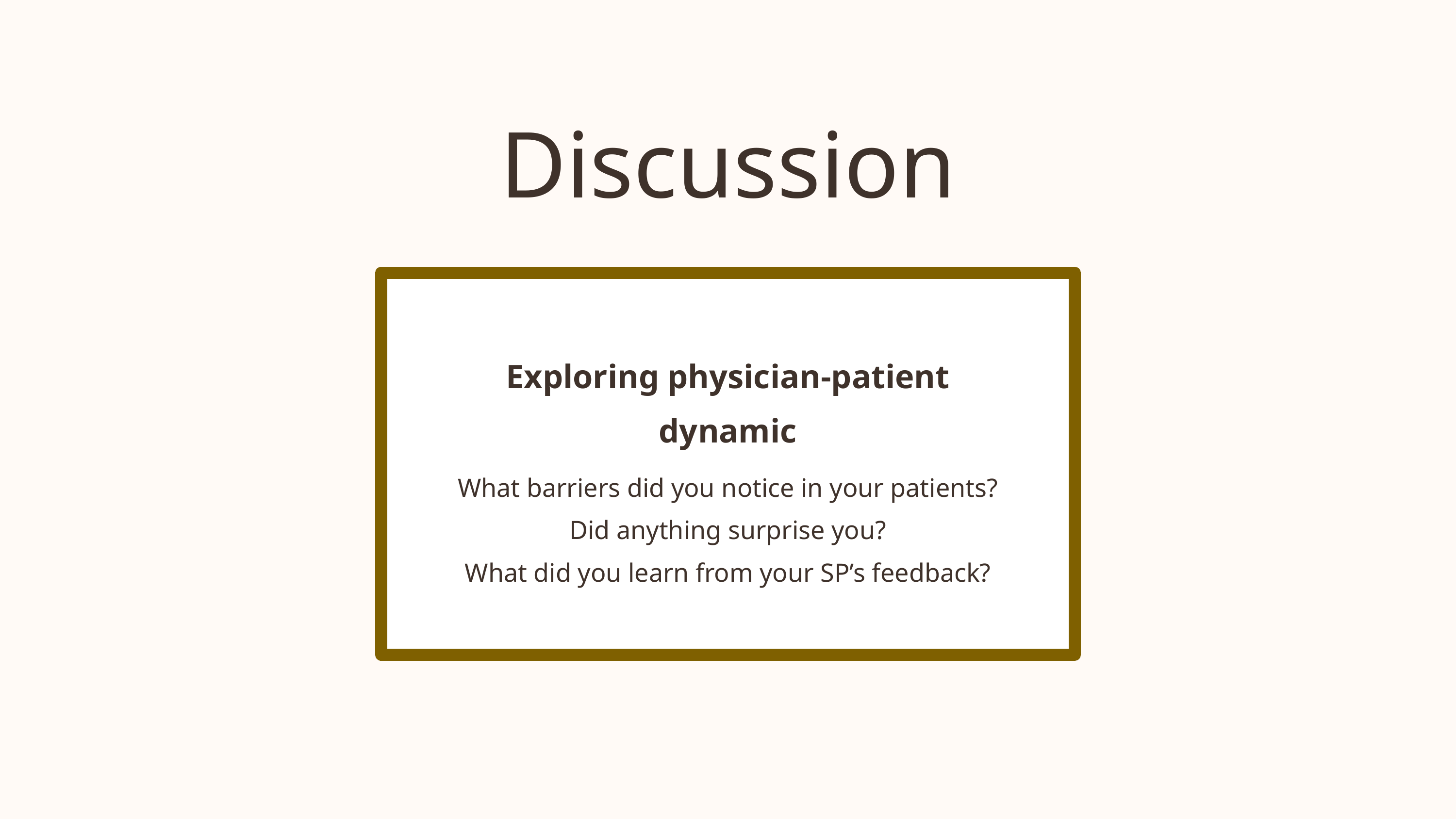

Discussion
Exploring physician-patient dynamic
What barriers did you notice in your patients?
Did anything surprise you?
What did you learn from your SP’s feedback?

## Slide 3
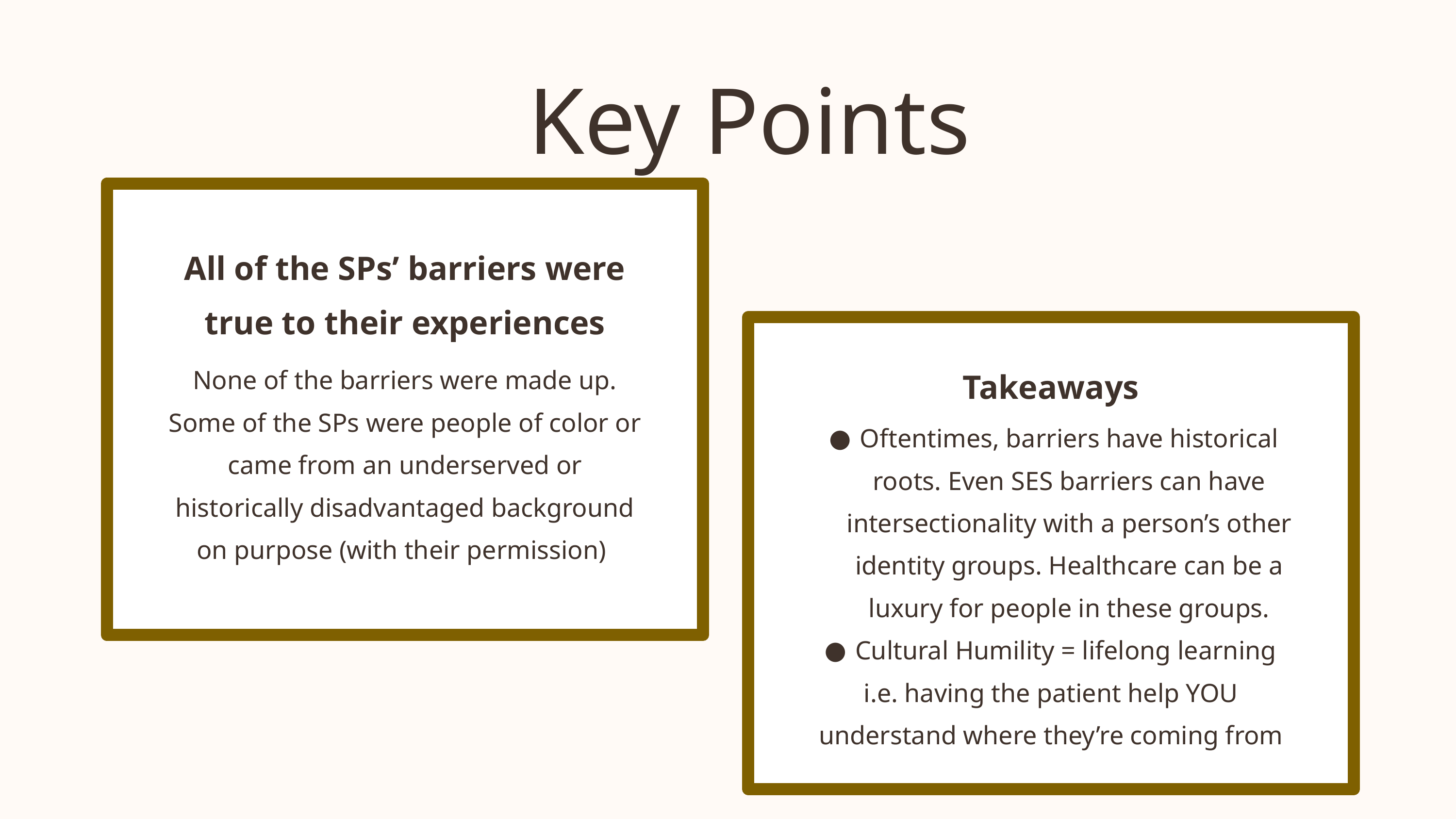

Key Points
All of the SPs’ barriers were true to their experiences
Takeaways
None of the barriers were made up.
Some of the SPs were people of color or came from an underserved or historically disadvantaged background on purpose (with their permission)
Oftentimes, barriers have historical roots. Even SES barriers can have intersectionality with a person’s other identity groups. Healthcare can be a luxury for people in these groups.
Cultural Humility = lifelong learning
i.e. having the patient help YOU understand where they’re coming from

## Slide 4
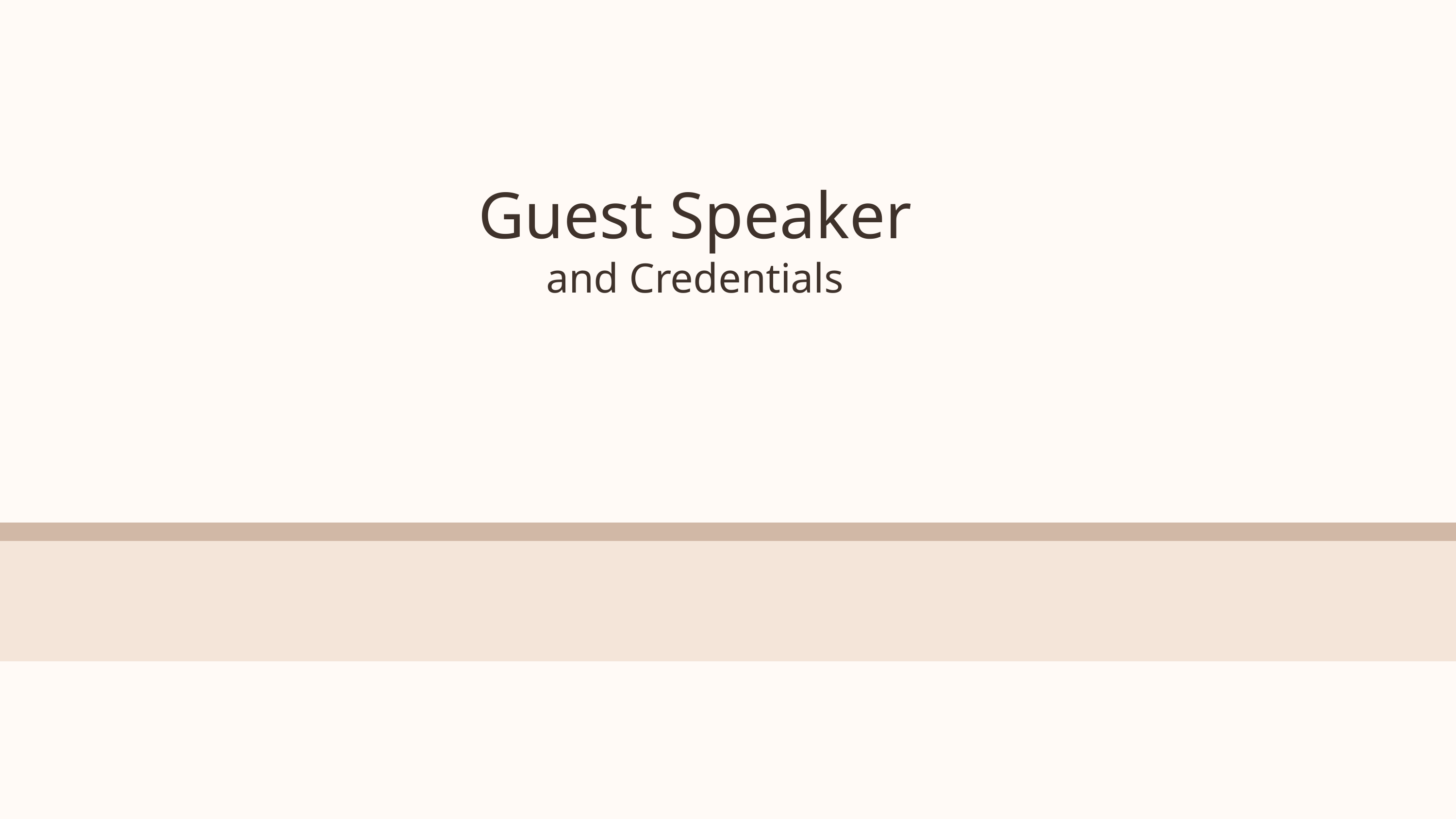

Guest Speaker
and Credentials

## Slide 5
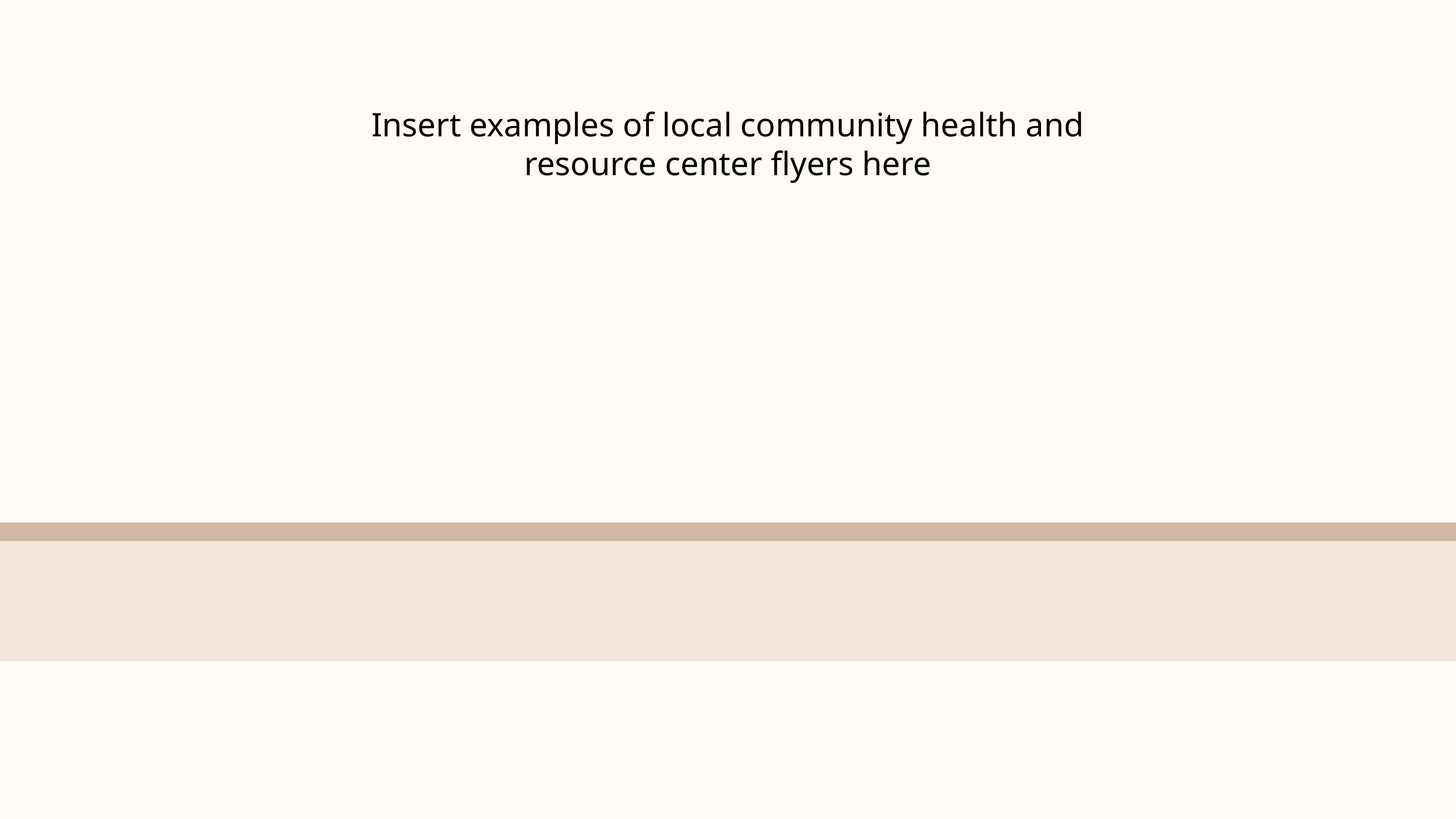

Insert examples of local community health and resource center flyers here

## Slide 6
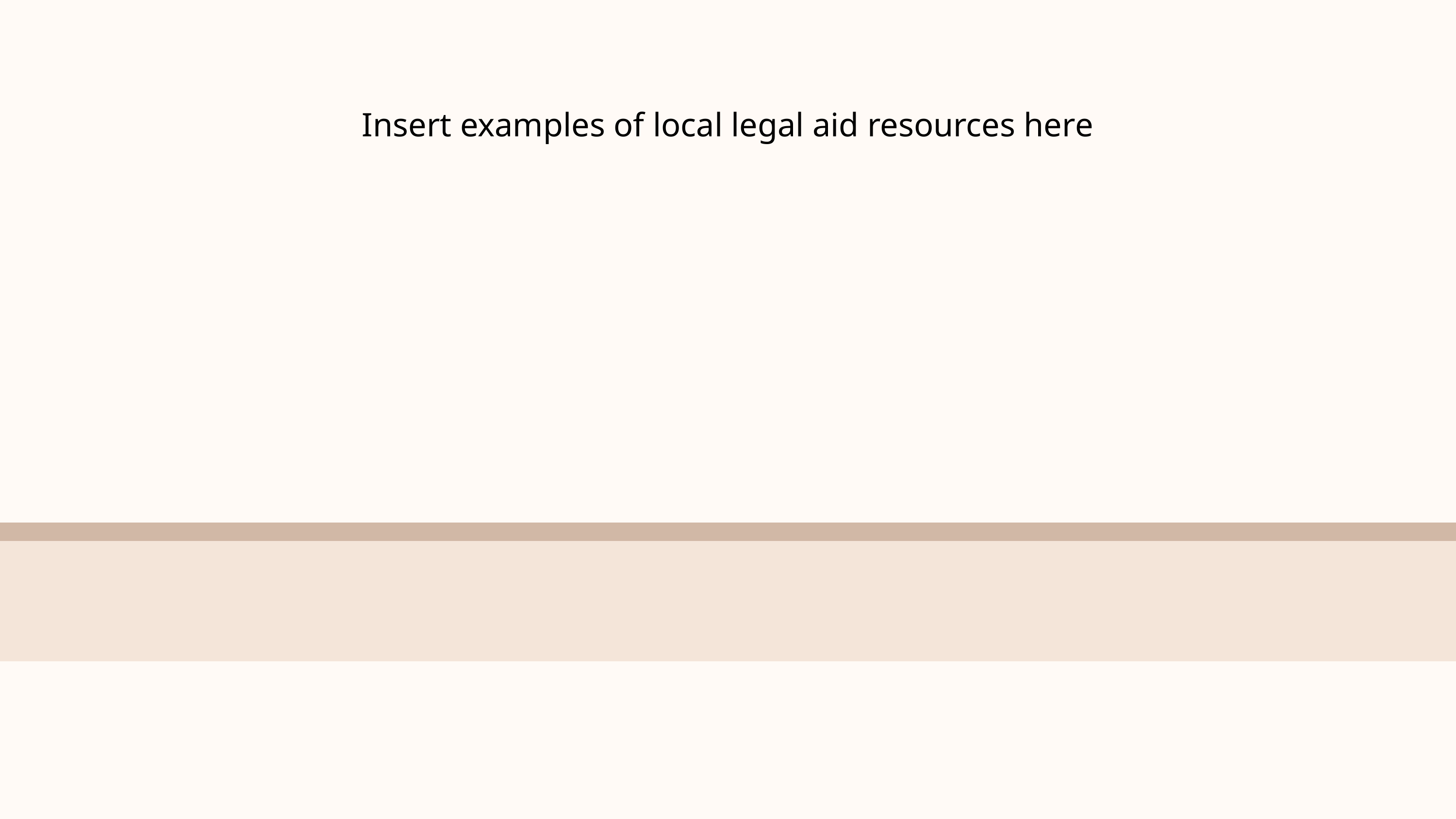

Insert examples of local legal aid resources here

## Slide 7
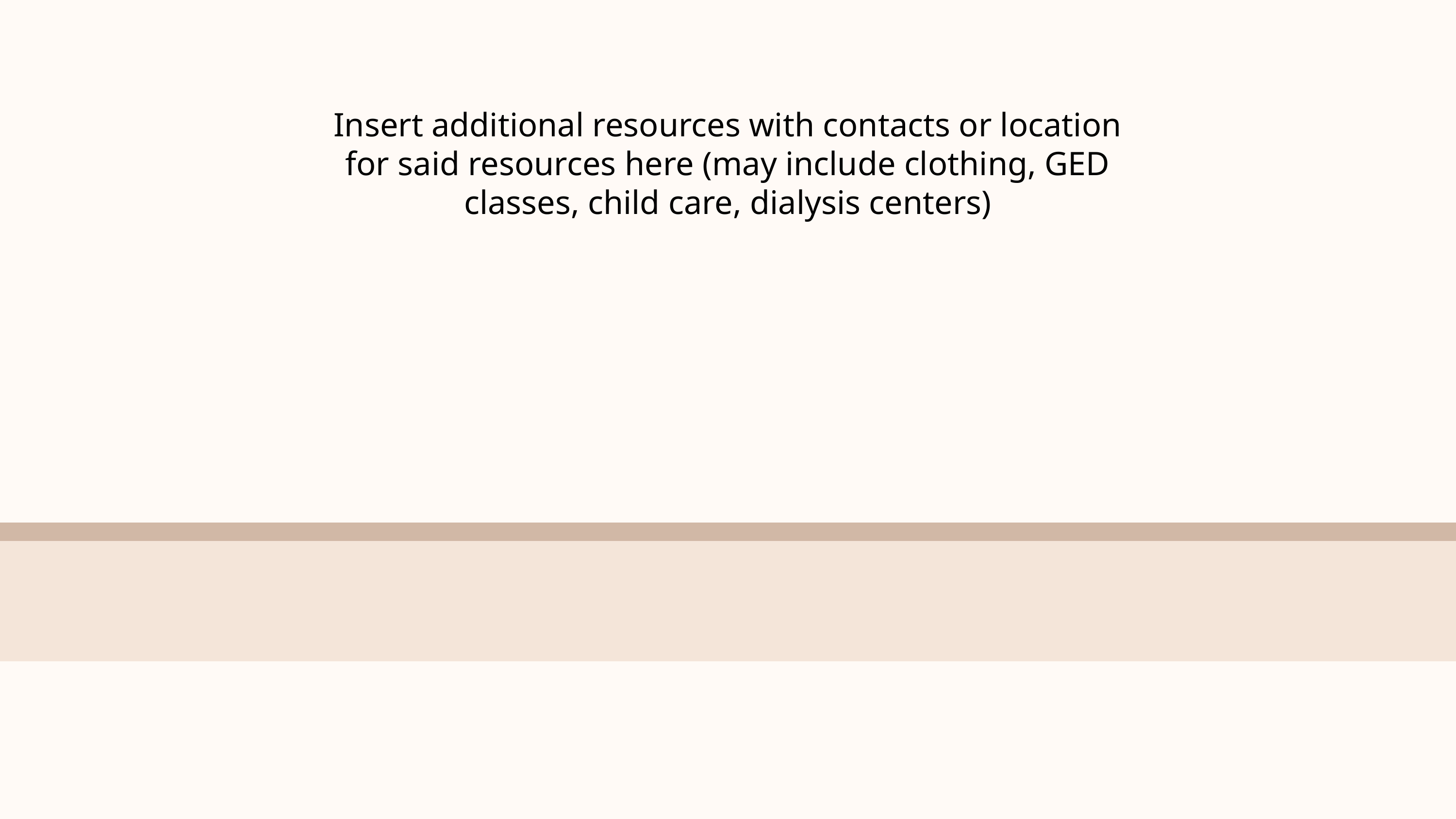

Insert additional resources with contacts or location for said resources here (may include clothing, GED classes, child care, dialysis centers)

## Slide 8
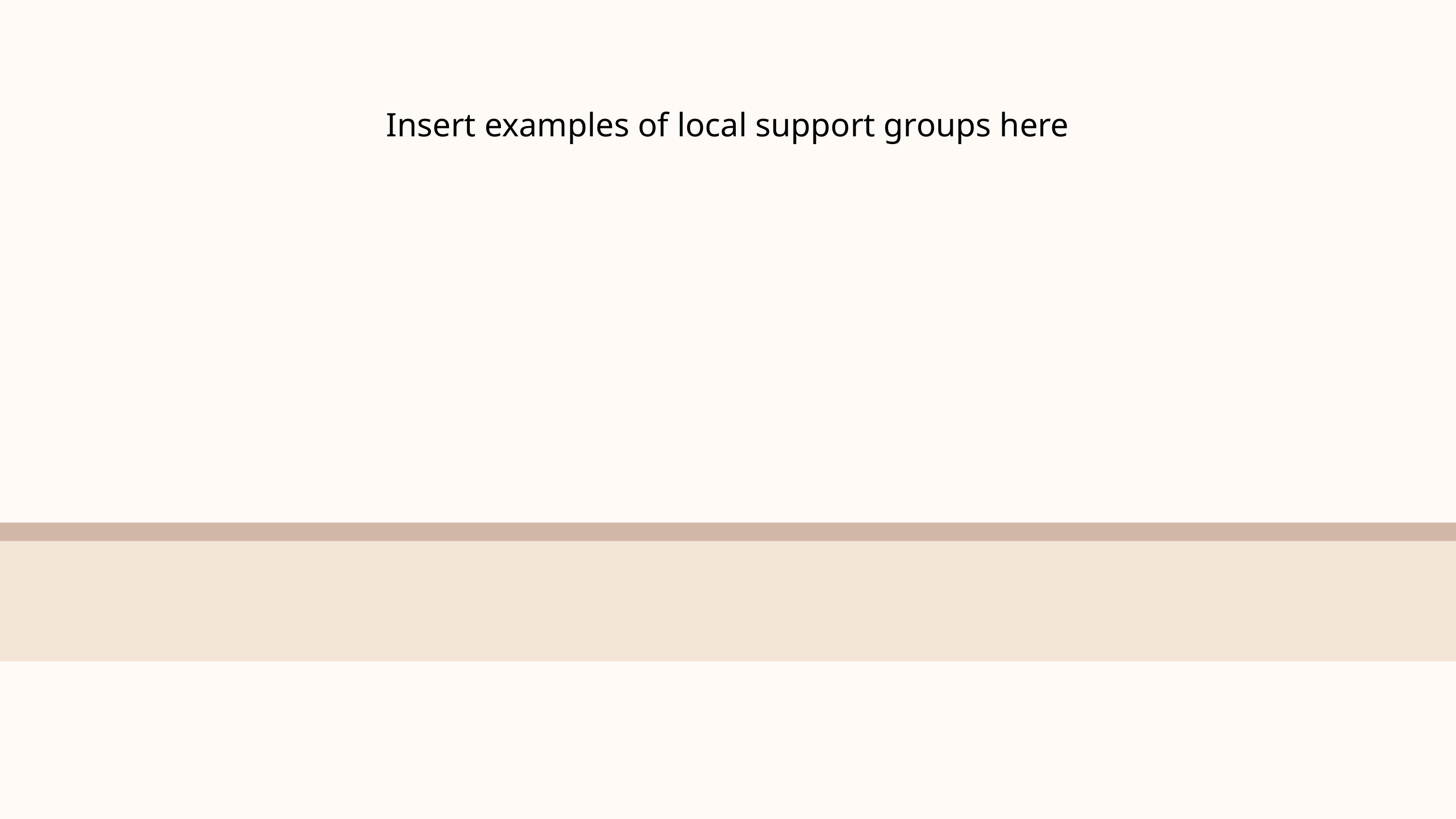

Insert examples of local support groups here

## Slide 9
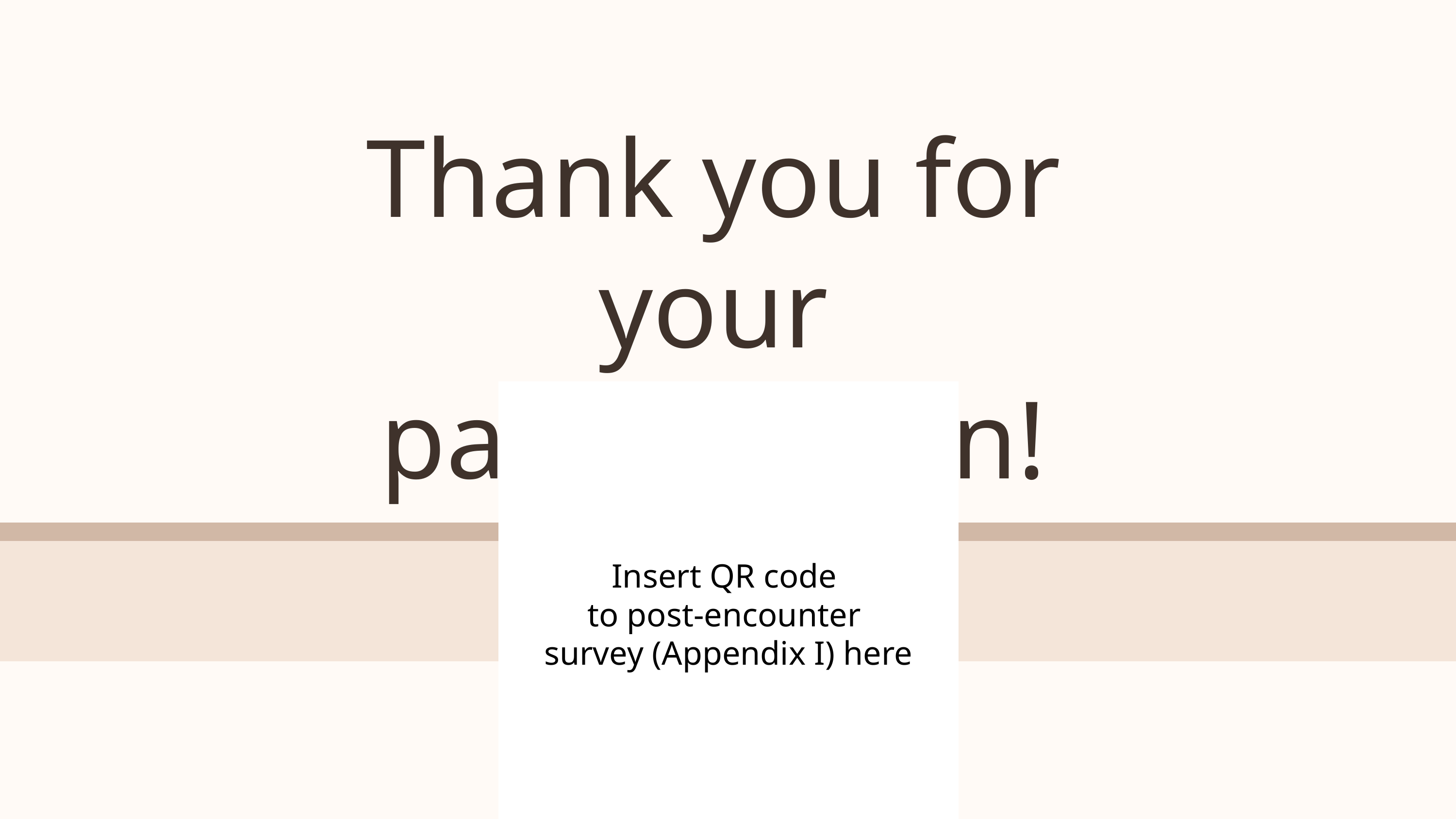

Thank you for your participation!
Insert QR code
to post-encounter
survey (Appendix I) here
