# Supplementary material for: Barriers to Care Encounter: A Model That Empowers Underserved Populations and Promotes Cross-Cultural Preparedness in Medical Students
Source: MedEdPORTAL. 2026 Jun 11;22:11608. doi: 10.15766/mep_2374-8265.11608 (PMC13253653; doi:10.15766/mep_2374-8265.11608)
Supplement: Supplementary file 1 — SP Case.docxLecture and Prebrief.pptxStudent Preencounter Instructions.docxStudent Guide for Gathering a History.docxPreencounter Survey.docxCommunication Skills Checklist.docxDebrief Discussion Questions.docxPostencounter Debrief Presentation.pptxPostencounter Survey.docxRecruitment Flyer.docxCase Overview and SP Training.docx [file mep_2374-8265.11608-s001.zip › I. Postencounter Survey.docx]

Barriers to Care Encounter Post-Survey

Please fill out this anonymous survey to allow us to evaluate this new standardized patient encounter.

1. Please enter the last 5 digits of your cell phone number. (Ex. 11111)

Note: Data will not be traced to you. These digits will be used to track improvement anonymously and determine the helpfulness of the encounter.

1. Today's Date (MM/DD/YY)
2. Select your level of agreement with the following statements:

(1=Strongly Disagree, 2=Slightly Disagree, 3=Neither Agree nor Disagree, 4=Slightly Agree, 5=Strongly Agree)

- 1. I can identify the historical impact of racism and cultural issues in relation to current health disparities.
  2. I can describe systemic and medical encounter issues.
  3. I can elicit a cultural, social, and medical history.
  4. I can assess and enhance patient adherence.
  5. I value and wish to learn how to address social determinants of health (including safe housing/neighborhoods, transportation, racism, discrimination, violence, education, job opportunities, income, access to nutritious foods, physical activity opportunities, polluted air and water, and/or language and literacy skills).
  6. I understand how my cultural background and biases influence my interactions with people from different backgrounds.
  7. This was a valuable learning experience.

1. Do you have any comments or feedback on this encounter?
