# Supplementary material for: Barriers to Care Encounter: A Model That Empowers Underserved Populations and Promotes Cross-Cultural Preparedness in Medical Students
Source: MedEdPORTAL. 2026 Jun 11;22:11608. doi: 10.15766/mep_2374-8265.11608 (PMC13253653; doi:10.15766/mep_2374-8265.11608)
Supplement: Supplementary file 1 — SP Case.docxLecture and Prebrief.pptxStudent Preencounter Instructions.docxStudent Guide for Gathering a History.docxPreencounter Survey.docxCommunication Skills Checklist.docxDebrief Discussion Questions.docxPostencounter Debrief Presentation.pptxPostencounter Survey.docxRecruitment Flyer.docxCase Overview and SP Training.docx [file mep_2374-8265.11608-s001.zip › J. Recruitment Flyer.docx]

Volunteers Needed

Barriers to Care Patient Encounter

We want to help medical students communicate better with patients from different cultural backgrounds who've experienced barriers to healthcare

WE NEED VOLUNTEERS WHO'VE

EXPERIENCED BARRIERS SUCH AS:

- Distrust of healthcare system
- Doctor did not understand your cultural beliefs
- Doctor did not understand your religious beliefs
- Lack of transportation to appointments/specialists/labs
- Cost of care/lab tests
- Insufficient insurance coverage
- Literacy level
- Inability to take time off work

VOLUNTEERS WILL BE PRACTICE PATIENTS

AND GIVEN VOLUNTEER HOURS

If you've experienced any of these barriers, please join us

at *LOCATION* on *DATE*

To sign up, please visit *link* or scan this QR code:

*insert QR code here*
