# Supplementary material for: Barriers to Care Encounter: A Model That Empowers Underserved Populations and Promotes Cross-Cultural Preparedness in Medical Students
Source: MedEdPORTAL. 2026 Jun 11;22:11608. doi: 10.15766/mep_2374-8265.11608 (PMC13253653; doi:10.15766/mep_2374-8265.11608)
Supplement: Supplementary file 1 — SP Case.docxLecture and Prebrief.pptxStudent Preencounter Instructions.docxStudent Guide for Gathering a History.docxPreencounter Survey.docxCommunication Skills Checklist.docxDebrief Discussion Questions.docxPostencounter Debrief Presentation.pptxPostencounter Survey.docxRecruitment Flyer.docxCase Overview and SP Training.docx [file mep_2374-8265.11608-s001.zip › K. Case Overview and SP Training.docx]

**Patient Training**

- **Volunteers are free to bring in a copy of this form if they want to so they don’t have to memorize this information**
- **Student = medical student**
- **Standardized patient or SP or patient = you**

| **Case Overview (Hypertension)** | |
| --- | --- |
| **Patient Name, Age, Gender** | Maya/Michael Willow, Age is SP’s age, Gender is SP’s gender. Pronouns are SP’s pronouns. |
| **Presenting Situation** | Patient reporting to visit for a hypertension (high blood pressure) follow-up |
| **Psychosocial Profile** | Patient should be tired and slightly uncomfortable/standoffish at first due to prior dissatisfaction with the healthcare system/previous providers |
| **Opening Statement/ Chief Complaint** | What specifically is the standardized patient going to tell the student in response to “What brings you here today?”:  “Here to check my blood pressure and breathing” |
| **Open-Ended Questions** | SP response to the open-ended question of “What brings you here today?” and “Tell me more about that?”  Came in for a check up with a different doctor last year and was told my blood pressure was really high and now I’m having bad headaches, and a hard time breathing.  If asked, patient does not check their blood pressure at home. |
| **History of Present Illness** | **· Onset (when did it start?):**  I had a blood pressure of 160/90 about a year ago but the headaches, and difficulty breathing started this week.  **· Position:**  Headaches: both temples  **· Radiation (Does the pain go anywhere?):**  No radiation, the pain just stays around my temples.  **· Quality:**  The headaches feel like a sharp stabbing pain  Hard to take deep breaths/get enough air in  **· Quantification:**  Headache pain is 7/10.  It’s been hard to breathe all week but sometimes it feels like a 2/10 and other times it feels like a 5/10. It just depends on the day. Today it feels like a 4/10.  All pain occurs every day and never goes away (constant)  **· Related symptoms:**  Some dull chest pain. No coughing, wheezing, fevers, or chills. Has not had an A1C/blood sugar level checked recently.  **· Setting:**  Nothing has changed recently and no recent illness.  Not checking blood pressure at home.  **· Transforming factors (Does anything make it better or worse?):**  Nothing makes it feel better. Tried Tylenol and Ibuprofen.  It gets worse when I’m walking or while at work.  Patient response to “how is this affecting your life?”  It makes it hard to focus or concentrate on anything and now I’m getting worried that something’s really wrong with my chest. |
| **Medications** | · Prescriptions: I was prescribed Lisinopril, Metformin, and Jardiance but I’m not taking them.  (If asked why you’re not taking the meds at this point, respond with I know I’m supposed to take them, but I just don’t. For now, I just wanna give you my history and hear about my blood pressure and breathing)  · Over The Counter (OTC): none  · Herbs/supplements: none |
| **Allergies** | · Drugs: None  · Food: None |
| **Drugs, Alcohol, Tobacco, Caffeine** | · Alcohol: None  · Drugs: None  · Smoking: Never  · Caffeine: None |
| **Past Medical History** | Childhood: none  Adult: Type II Diabetes, hypertension  If asked, no history of thyroid, cortisol, or aldosterone issues  Injuries/Accidents: none  Hospitalizations: none  Surgeries/Procedures: None  Psychiatric: None  Immunizations: Up To Date (UTD)  OB/Gyn (if age >45): No previous pregnancies or live births. Last menstrual period was over a year ago. Age of Menopause was 45. No related symptoms.  OB/Gyn (if age 18 - 45): No previous pregnancies or live births. Last menstrual period was 2 weeks ago. Periods are regular and monthly, with some cramps. |
| **Social History** | Birthplace: Lubbock, Texas  Last level of education completed: High school  Who is in their household: Living in an apartment with Spouse (Happy with your partner)  Occupation: Works at Market Street.  Does SP live in a safe environment? Yes  Hobbies: [Answer with your hobbies]  Sexual history: 5 lifetime partners, sexually active with spouse, uses contraceptives, no history of STIs, has experienced vaginal sex  Diet: Sandwich or salad for lunch, and a healthy dinner.  Exercise: walks around neighborhood |
| **Barriers**  *(These are suggestions*  *for initial responses)* | Student asks, “Is there anything going on in your life that’s making it hard for you to take your medications?” or “…any barriers preventing you from taking your medications?”  **SP can elaborate or replace with details using their lived experience with a barrier to care***  (Distrust) I grew up in a small town where receiving adequate healthcare was a luxury. My parents and grandparents would tell me stories that sparked my distrust. But now I’ve experienced it myself. With my last doctor, I’d always have to be the one advocating for myself and asking for scans to be done or offering my family history because he didn’t take initiative to ask. I always felt like my pain was minimized or met with little to no treatment, so I left/stopped going and my blood pressure medications ran out.  (Inflexible work schedule) I just haven’t had time to go to all my doctors’ appointments. I’m working paycheck to paycheck 7 days a week so I’ve missed a few follow-ups.  (Cost of care) I’m paying my way through school so I can’t afford to get more medications right now. I just pay for my insulin.  (Insurance) My insurance doesn’t cover the full price of all of the medications.  (Transportation) I don’t have a car, so I have to get a ride to the clinic. There’s not always someone available.  (Cultural/language barrier) I came to my last appointment with my mother, who speaks *insert language here*, and it was difficult for us all to communicate. It was tough and saddening to see the doctors get frustrated and angry because of a language barrier, instead of trying to resolve the issue. So I’ve been waiting to get a new doctor, and I ran out of my blood pressure medications. |
| **Family Medical History** | Grandparents: Unknown  Mother: still alive (age = 30 years older than you), has hypertension  Father: still alive (age = 30 years older than you), has hypertension  No siblings  No children or pets |
| **Review of Systems (Past 2 weeks of symptoms)** | Student does not have to do full review of symptoms, but should look through ROS and ask about pertinent symptoms  SP positive for:  General: fatigue, weakness  Pulmonary: shortness of breath with sharp pain  Cardiovascular: Chest pain, Shortness of breath while lying flat, shortness of breath with exertion, swelling in legs up to the middle of the shin  Endocrine: Polydipsia (thirsty all the time) |
| **Physical Exam Findings:** | Height and weight in chart – 5’ 10” and 190 lbs.  Vitals: BP: **176/99 mm Hg.** Students can obtain heart and respiratory rate if they would like practice.  Student does not have to listen to heart and lungs, but they can if they would like practice, findings normal |
| **Plan/Support** | Student should end the encounter by offering support/resources for the patient’s barrier and reemphasizing a desire to help the patient achieve their goals |
| **Special Instructions:** | Patient should give little to no detail about personal barriers until after the social history if the student has made them feel comfortable and heard |

## *For this encounter, Standardized Patients (SPs) are selected to participate if they have in real life experienced a barrier to their own healthcare that they are willing to act out in this simulated encounter. Specific barriers that can be used for this encounter include but are not limited to: cost of care, insurance coverage, lack of transportation, inflexible work schedule, distrust in the healthcare system (prior negative experiences with the system/providers), and language/cultural barriers.

##

## Medical Student Pre-Encounter Instructions

| **Station Name:** | **“**Maya/Michael Willow**”** |
| --- | --- |
| **Purpose of Case:** | - Demonstrate patient-centered communication skills using Motivational Interviewing (OARS Model)  - Obtain full health history and targeted ROS  - Establish Rapport by getting to know your patient and facilitating a values-based conversation  - Suggest a treatment plan |
| **Setting:** | Family Clinic |
| **Presenting Situation (Reason for Visit):** | Hypertension |
| **Vital Signs:** | 5’10” and 190 lbs. (May not match SP)  Obtain other vitals if you would like practice |
| **Allergies:** | Obtain |
| **Medications:** | Obtain |
| **ROS:** | Ask pertinent symptoms only (General, Cardiovascular, Respiratory, Endocrine) |
| **Physical Exam:** | Student may listen to Heart and Lungs, but does not have to |

**INSTRUCTIONS TO STUDENT:**

**1.** **You will have 15 minutes to take a focused medical history.**

**2.** **The standardized patient will take about 5 minutes to provide verbal feedback.**

**3. You will have a debrief session afterwards.**

*[This should include instructions for any post-encounter activities (i.e., SOAP note, questions, etc.) that should be completed by the student.]*

**Patient Training**

- **Volunteers are free to bring in a copy of this form if they want to so they don’t have to memorize this information**
- **Student = medical student**
- **Standardized patient or SP or patient = you**

| **Case Overview (Asthma)** | |
| --- | --- |
| **Patient Name, Age, Gender** | Maya/Michael Willow, Age is SP’s age, Gender is SP’s gender. Pronouns are SP’s pronouns. |
| **Presenting Situation** | Patient reporting to visit for difficulty breathing |
| **Psychosocial Profile** | Patient should be tired and slightly uncomfortable/standoffish at first due to prior dissatisfaction with the healthcare system/previous providers |
| **Opening Statement/ Chief Complaint** | What specifically is the standardized patient (or SP, which is you) going to tell the student in response to “What brings you here today?”:  “Hard time breathing” |
| **Open-Ended Questions** | SP response to the open-ended question of “What brings you here today?” and “Tell me more about that?”  I’ve felt short of breath all week. I’ve felt like this before but it’s never lasted this long before. Sometimes, I’ll just be laughing and start coughing or wheezing. |
| **History of Present Illness** | **· Onset (when did it start?):**  I’ve had breathing issues since I was little (age 7) but it feels like it started getting worse about a week ago.  **· Position (where is the pain?):**  No chest pain but chest feels tight all over (on both sides).  **· Radiation (Does the pain go anywhere?):**  No chest pain. Tightness just stays in the whole chest.  **· Quality (can you describe it?):**  Chest tightness all over.  Cough is dry. Not coughing anything up.  Hard to take deep breaths and hard to breathe out specifically.  **· Quantification:**  Chest tightness is 4/10.  Cough is 6/10.  Coughs and wheezes about 1-2x/day. The trouble breathing used to come and go, but it’s been constant all week.  **· Related symptoms:**  Some wheezing, dry cough. No fevers, chills, sore throat, nasal congestion.  **· Setting:**  Nothing has changed recently and no recent illness.  **· Transforming factors (Does anything make it better or worse?):**  Nothing makes it feel better. Breathing gets worse when I’m walking/exercising and it gets worse at night. Also gets worse when walking outside in the cold.  Patient response to “how is this affecting your life?”  It makes it hard to focus or concentrate on anything and now I’m getting worried that something’s really wrong with my lungs. |
| **Medications** | · Prescriptions: I’m taking insulin (Humalog) every day 15 minutes before meals. I used to have an inhaler but I don’t have one right now.  (If asked why you’re not using an inhaler at this point, respond with I know I’m supposed to use one, but I just don’t. For now, I just wanna give you my history and hear about my breathing)  · Over The Counter (OTC): none  · Herbs/supplements: none |
| **Allergies** | · Drugs: None  · Food: None |
| **Drugs, Alcohol, Tobacco, Caffeine** | · Alcohol: None  · Drugs: None  · Smoking: Never  · Caffeine: None |
| **Past Medical History** | Childhood: Asthma  Adult: Type I Diabetes  If asked, no history of thyroid, cortisol, or aldosterone issues  Injuries/Accidents: none  Hospitalizations: none  Surgeries/Procedures: None  Psychiatric: None  Immunizations: Up To Date (UTD)  OB/Gyn (if age >45): No previous pregnancies or live births. Last menstrual period was over a year ago. Age of Menopause was 45. No related symptoms.  OB/Gyn (if age 18 - 45): No previous pregnancies or live births. Last menstrual period was 2 weeks ago. Periods are regular and monthly, with some cramps. |
| **Social History** | Birthplace: [Use your birthplace]  Living situation: Apartment in Lubbock, Texas  Who is in their household: College Roommate  Do they live in a safe environment? Yes  Last level of education completed: High school, At TTU now  [can share your Major if asked]  Occupation: Works at Follett Bookstore on Campus  Hobbies: [Answer with your hobbies]  Sexual history: sexually active, 1 lifetime partner, uses contraceptives, no history of STIs, has experienced vaginal sex (answer can be “no intimate sexual experiences” if contradictory with religion)  Diet: Sandwich or salad for lunch, and a healthy dinner.  Exercise: walks around campus, occasionally goes to the Rec |
| **Barriers**  *(These are suggestions*  *for initial responses)* | Student asks, “Is there anything going on in your life that’s making it hard for you to take your medications?” or “…any barriers preventing you from taking your medications?”  **SP can elaborate or replace with details using their lived experience with a barrier to care***  (Distrust) I grew up in a small town where receiving adequate healthcare was a luxury. My parents and grandparents would tell me stories that sparked my distrust. But now I’ve experienced it myself. With my last doctor, I’d always have to be the one advocating for myself and asking for scans to be done or offering my family history because he didn’t take initiative to ask. I always felt like my pain was minimized or met with little to no treatment, so I left/stopped going and my blood pressure medications ran out.  (Inflexible work schedule) I just haven’t had time to go to all my doctors’ appointments. I’m working paycheck to paycheck 7 days a week so I’ve missed a few follow-ups.  (Cost of care) I’m paying my way through school so I can’t afford to get more medications right now. I just pay for my insulin.  (Insurance) My insurance doesn’t cover the full price of all of the medications.  (Transportation) I don’t have a car, so I have to get a ride to the clinic. There’s not always someone available.  (Cultural/language barrier) I came to my last appointment with my mother, who speaks *insert language here*, and it was difficult for us all to communicate. It was tough and saddening to see the doctors get frustrated and angry because of a language barrier, instead of trying to resolve the issue. So I’ve been waiting to get a new doctor, and I ran out of my blood pressure medications. |
| **Family Medical History** | Grandparents: Unknown  Mother: still alive (age = 30 years older than you), has asthma  Father: still alive (age = 30 years older than you), has asthma  No siblings  No children or pets |
| **Review of Systems (Past 2 weeks of symptoms)** | Student does not have to do full review of symptoms, but should look through ROS and ask about pertinent symptoms  SP positive for:  Pulmonary: shortness of breath, wheezing, dry cough  Endocrine: Polydipsia (thirsty all the time) |
| **Physical Exam Findings:** | Height and weight in chart – 5’ 10” and 190 lbs.  Vitals: BP: **116/79 mm Hg.** Students can obtain heart and respiratory rate if they would like practice.  Student does not have to listen to heart and lungs, but they can if they would like practice, findings: wheezes bilaterally (both lungs) |
| **Plan/Support** | Student should end the encounter by offering support/resources for the patient’s barrier and reemphasizing a desire to help the patient achieve their goals |
| **Special Instructions:** | Patient should give little to no detail about personal barriers until after the social history if the student has made them feel comfortable and heard |

## *For this encounter, Standardized Patients (SPs) are selected to participate if they have in real life experienced a barrier to their own healthcare that they are willing to act out in this simulated encounter. Specific barriers that can be used for this encounter include but are not limited to: cost of care, insurance coverage, lack of transportation, inflexible work schedule, distrust in the healthcare system (prior negative experiences with the system/providers), and language/cultural barriers.

##

## Medical Student Pre-Encounter Instructions

| **Station Name:** | **“**Maya/Michael Willow**”** |
| --- | --- |
| **Purpose of Case:** | - Demonstrate patient-centered communication skills using Motivational Interviewing (OARS Model)  - Obtain full health history and targeted ROS  - Establish Rapport by getting to know your patient and facilitating a values-based conversation  - Suggest a treatment plan |
| **Setting:** | Family Clinic |
| **Presenting Situation (Reason for Visit):** | Asthma |
| **Vital Signs:** | 5’10” and 190 lbs. (May not match SP)  Obtain other vitals if you would like practice |
| **Allergies:** | Obtain |
| **Medications:** | Obtain |
| **ROS:** | Ask pertinent symptoms only (General, Cardiovascular, Respiratory, Endocrine) |
| **Physical Exam:** | Student may listen to Heart and Lungs, but does not have to |

**INSTRUCTIONS TO STUDENT:**

**1.** **You will have 15 minutes to take a focused medical history.**

**2.** **The standardized patient will take about 5 minutes to provide verbal feedback.**

**3. You will have a debrief session afterwards.**

*[This should include instructions for any post-encounter activities (i.e., SOAP note, questions, etc.) that should be completed by the student.]*

**OARS Model for Motivational Interviewing for Medical Students to Reference**

- Asks 3 **open** ended questions (p. 41 SAMHSA MI) only some applicable
  - What brings you in today?
  - Tell me more about
  - Tell me when…
  - Tell me what it’s like when…
  - How is this affecting your life?
  - What do you want to do about____?
  - How would you like to go about this/how would you like things to change?
  - How would you like your life to be different a year from now?
  - What needs to happen?
  - Barriers to care: “Are there any barriers that make it hard to get or take your medications?”
- **Affirming** (might be case by case) (framing statements with “you”)
  - You took a big step in coming here today
  - Coming here was hard, but you did it
  - Going through that is hard, but you did it
  - (For follow-ups) you’ve been working hard
  - Things didn’t turn out how you wanted, but you tried really hard
- **Reflective** listening (student restated/rephrased SP’s responses. Can focus SP’s feelings)
- **Summarizing** (student repeats big picture to SP before leaving. Students select statements that had meaning to the SP such as SP’s values/desires. Emphasizes “change talk” if applicable)
  - Asks if they missed anything
